# Supplementary figures and images for: Diversity of nutritional content in seeds of Brazilian common bean germplasm
Source: PLoS One. 2020 Sep 28;15(9):e0239263. doi: 10.1371/journal.pone.0239263 (PMC7521705; doi:10.1371/journal.pone.0239263)

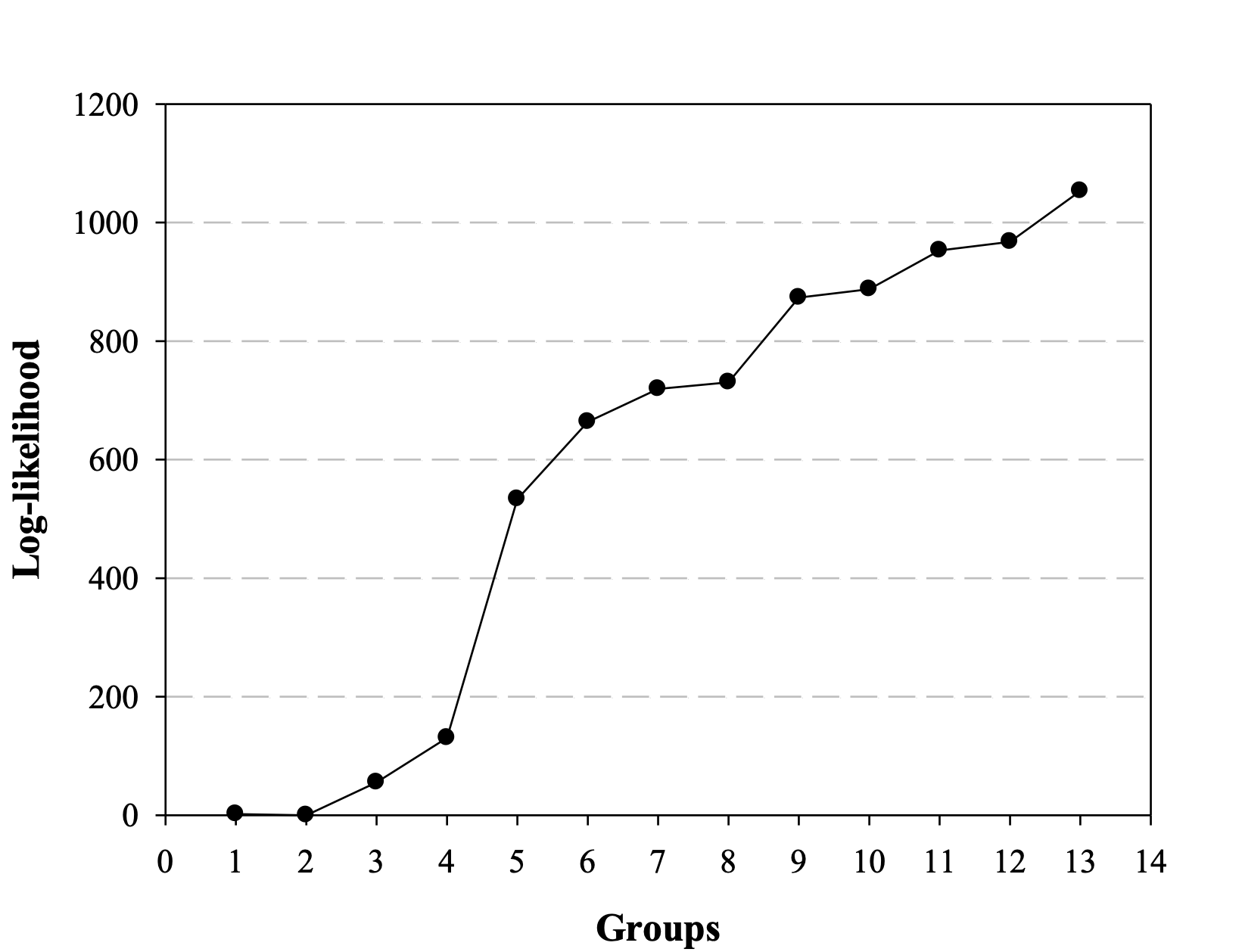

Supplement: S1 Fig — (TIF) [file pone.0239263.s001.tif]
