# Supplementary material for: Diversity of nutritional content in seeds of Brazilian common bean germplasm
Source: PLoS One. 2020 Sep 28;15(9):e0239263. doi: 10.1371/journal.pone.0239263 (PMC7521705; doi:10.1371/journal.pone.0239263)
Supplement: S1 Table — 1CIAT = International Center for Tropical Agriculture (Centro Internacional de Agricultura Tropical), EMBRAPA = Brazilian Agricultural Research Corporation (Empresa Brasileira de Pesquisa Agropecuária), IAC = Agronomic Institute of Campinas (Instituto Agronômico de Campinas), IDR-Paraná = Rural Development Institute of Paraná –IAPAR–EMATER (Instituto de desenvolvimento Rural do Paraná), USDA = United States Department of Agriculture, ESALQ = Universidade de São Paulo—Escola Superior de Agricultura “Luiz de Queiroz”. (DOCX) [file pone.0239263.s002.docx]

**S1 Table. Information of the 1,512 accessions characterized for seed nutritional content.**

| **Accessions** | **Developing institution**^1^ | **Genetic Material** | **Seed coat color** | **Origin** | **Ward-MLM Group** |
| --- | --- | --- | --- | --- | --- |
| A210 | CIAT | Breeding Lines | Brown - (Colors) | Mesoamerican | G2 |
| A248 | CIAT | Breeding Lines | Yellow - (Colors) | Mesoamerican | G2 |
| A283 | CIAT | Breeding Lines | Red - (Colors) | Mesoamerican | G5 |
| A295 | CIAT | Breeding Lines | Brown - (Colors) | Mesoamerican | G4 |
| A445 | CIAT | Breeding Lines | Brown - (Colors) | Mesoamerican | G5 |
| A483 | CIAT | Breeding Lines | Brown - (Colors) | Andean | G2 |
| A520 | CIAT | Breeding Lines | Brown - (Colors) | Mesoamerican | G3 |
| A525 | CIAT | Breeding Lines | Brown - (Colors) | Mesoamerican | G2 |
| A531 | CIAT | Breeding Lines | Brown - (Colors) | Mesoamerican | G4 |
| A553 | CIAT | Breeding Lines | Brown - (Colors) | Mesoamerican | G4 |
| A563 | CIAT | Breeding Lines | Brown - (Colors) | Mesoamerican | G4 |
| A564 | CIAT | Breeding Lines | Brown - (Colors) | Mesoamerican | G2 |
| A566 | CIAT | Breeding Lines | Brown - (Colors) | Mesoamerican | G3 |
| A567 | CIAT | Breeding Lines | Brown - (Colors) | Mesoamerican | G3 |
| A576 | CIAT | Breeding Lines | Brown - (Colors) | Mesoamerican | G2 |
| A580 | CIAT | Breeding Lines | Yellow - (Colors) | Mesoamerican | G2 |
| A589 | CIAT | Breeding Lines | Brown - (Colors) | Mesoamerican | G3 |
| A59 | CIAT | Breeding Lines | Brown - (Colors) | Mesoamerican | G4 |
| A604 | CIAT | Breeding Lines | Brown - (Colors) | Mesoamerican | G4 |
| A623 | CIAT | Breeding Lines | Brown - (Colors) | Mesoamerican | G2 |
| A649 | CIAT | Breeding Lines | Brown - (Colors) | Mesoamerican | G5 |
| A663 | CIAT | Breeding Lines | White - (Colors) | Mesoamerican | G2 |
| A664 | CIAT | Breeding Lines | Yellow - (Colors) | Mesoamerican | G5 |
| A672 | CIAT | Breeding Lines | Brown - (Colors) | Mesoamerican | G5 |
| A673 | CIAT | Breeding Lines | Brown - (Colors) | Mesoamerican | G2 |
| A674 | CIAT | Breeding Lines | Red - (Colors) | Mesoamerican | G5 |
| A676 | CIAT | Breeding Lines | Red - (Colors) | Mesoamerican | G5 |
| A680 | CIAT | Breeding Lines | Brown - (Colors) | Mesoamerican | G5 |
| A681 | CIAT | Breeding Lines | Pinto - (Colors) | Mesoamerican | G5 |
| A682 | CIAT | Breeding Lines | Pinto - (Colors) | Mesoamerican | G5 |
| A687 | CIAT | Breeding Lines | Pinto - (Colors) | Mesoamerican | G5 |
| A690 | CIAT | Breeding Lines | Red - (Colors) | Mesoamerican | G3 |
| A705 | CIAT | Breeding Lines | Brown - (Colors) | Mesoamerican | G2 |
| A746 | CIAT | Breeding Lines | Red - (Colors) | Mesoamerican | G4 |
| A774 | CIAT | Breeding Lines | Red - (Colors) | Mesoamerican | G2 |
| A775 | CIAT | Breeding Lines | Brown - (Colors) | Mesoamerican | G2 |
| A779 | CIAT | Breeding Lines | Brown - (Colors) | Mesoamerican | G3 |
| A797 | CIAT | Breeding Lines | Brown - (Colors) | Mesoamerican | G3 |
| ABA34 | CIAT | Breeding Lines | White - (Colors) | Mesoamerican | G2 |
| Aete1/38 | IAC | Cultivars | Pink - (Colors) | Mesoamerican | G2 |
| Aete1/48 | IAC | Cultivars | White - (Colors) | Mesoamerican | G2 |
| Aete2 | IAC | Cultivars | Brown - (Colors) | Mesoamerican | G2 |
| AFR309 | CIAT | Breeding Lines | Pink - (Colors) | Andean | G1 |
| AFR311 | CIAT | Breeding Lines | Pink - (Colors) | Andean | G1 |
| AFR337 | CIAT | Breeding Lines | Pink - (Colors) | Mesoamerican | G2 |
| AFR345 | CIAT | Breeding Lines | Brown - (Colors) | Andean | G2 |
| Akitã | IAC | Cultivars | Brown - (Colors) | Mesoamerican | G2 |
| INT1 | Farmers | Landraces | Brown - (Colors) | Mesoamerican | G5 |
| AND673 | CIAT | Breeding Lines | Brown - (Colors) | Andean | G1 |
| AND710 | CIAT | Breeding Lines | Black Carioca - (Colors) | Andean | G2 |
| AND888 | CIAT | Breeding Lines | Purple - (Colors) | Andean | G1 |
| APG2921 | - | Breeding Lines | Purple - (Colors) | Mesoamerican | G2 |
| APG8919 | - | Breeding Lines | Yellow - (Colors) | Mesoamerican | G2 |
| APN108 | - | Breeding Lines | Purple - (Colors) | Mesoamerican | G2 |
| APN18 | - | Breeding Lines | Brown - (Colors) | Mesoamerican | G1 |
| APN82 | - | Breeding Lines | White - (Colors) | Mesoamerican | G4 |
| APN83 | - | Breeding Lines | Pinto - (Colors) | Mesoamerican | G4 |
| APN84A | - | Breeding Lines | Purple - (Colors) | Mesoamerican | G2 |
| APN93 | - | Breeding Lines | Brown - (Colors) | Mesoamerican | G2 |
| Aporé | Embrapa | Cultivars | Brown - (Colors) | Mesoamerican | G2 |
| ARA10 | CIAT | Breeding Lines | Brown - (Colors) | Mesoamerican | G1 |
| ARA13 | CIAT | Breeding Lines | Brown - (Colors) | Mesoamerican | G3 |
| ARC1 | CIAT | Breeding Lines | Brown - (Colors) | Mesoamerican | G2 |
| ARC2 | CIAT | Breeding Lines | Black Carioca - (Colors) | Mesoamerican | G3 |
| Line1 | IDR-Paraná | Breeding Lines | Brown - (Colors) | Mesoamerican | G1 |
| IAC Aruã | IAC | Cultivars | Brown - (Colors) | Mesoamerican | G2 |
| Aurora | - | Cultivars | Brown - (Colors) | Mesoamerican | G2 |
| Line2 | IDR-Paraná | Breeding Lines | Red - (Colors) | Mesoamerican | G3 |
| Line3 | IDR-Paraná | Breeding Lines | Purple - (Colors) | Mesoamerican | G3 |
| Line4 | IDR-Paraná | Breeding Lines | Brown - (Colors) | Mesoamerican | G2 |
| Line5 | IDR-Paraná | Breeding Lines | Purple - (Colors) | Mesoamerican | G2 |
| Line6 | IDR-Paraná | Breeding Lines | Brown - (Colors) | Mesoamerican | G3 |
| Line7 | IDR-Paraná | Breeding Lines | Brown - (Colors) | Mesoamerican | G2 |
| Line8 | IDR-Paraná | Breeding Lines | Brown - (Colors) | Mesoamerican | G2 |
| Line9 | IDR-Paraná | Breeding Lines | Brown - (Colors) | Mesoamerican | G2 |
| Line10 | IDR-Paraná | Breeding Lines | Red - (Colors) | Mesoamerican | G2 |
| Line11 | IDR-Paraná | Breeding Lines | Red - (Colors) | Mesoamerican | G3 |
| Line12 | IDR-Paraná | Breeding Lines | Brown - (Colors) | Mesoamerican | G1 |
| Line13 | IDR-Paraná | Breeding Lines | Brown - (Colors) | Mesoamerican | G2 |
| Line14 | IDR-Paraná | Breeding Lines | Red - (Colors) | Mesoamerican | G2 |
| Line15 | IDR-Paraná | Breeding Lines | Pinto - (Colors) | Mesoamerican | G2 |
| Line16 | IDR-Paraná | Breeding Lines | Pinto - (Colors) | Mesoamerican | G2 |
| Line17 | IDR-Paraná | Breeding Lines | Yellow - (Colors) | Mesoamerican | G2 |
| Line18 | IDR-Paraná | Breeding Lines | Pinto - (Colors) | Mesoamerican | G1 |
| Line19 | IDR-Paraná | Breeding Lines | Brown - (Colors) | Mesoamerican | G1 |
| Line20 | IDR-Paraná | Breeding Lines | Brown - (Colors) | Mesoamerican | G1 |
| Line21 | IDR-Paraná | Breeding Lines | Brown - (Colors) | Mesoamerican | G2 |
| Line22 | IDR-Paraná | Breeding Lines | Red - (Colors) | Mesoamerican | G2 |
| Line23 | IDR-Paraná | Breeding Lines | Brown - (Colors) | Mesoamerican | G3 |
| Line24 | IDR-Paraná | Breeding Lines | Brown - (Colors) | Mesoamerican | G2 |
| Line25 | IDR-Paraná | Breeding Lines | Brown - (Colors) | Mesoamerican | G2 |
| Line26 | IDR-Paraná | Breeding Lines | Brown - (Colors) | Mesoamerican | G3 |
| Line27 | IDR-Paraná | Breeding Lines | Brown - (Colors) | Mesoamerican | G3 |
| Line28 | IDR-Paraná | Breeding Lines | White - (Colors) | Mesoamerican | G3 |
| Line29 | IDR-Paraná | Breeding Lines | Red - (Colors) | Mesoamerican | G2 |
| Line30 | IDR-Paraná | Breeding Lines | Pinto - (Colors) | Mesoamerican | G3 |
| Line31 | IDR-Paraná | Breeding Lines | Brown - (Colors) | Mesoamerican | G3 |
| Line32 | IDR-Paraná | Breeding Lines | Red - (Colors) | Mesoamerican | G2 |
| Line33 | IDR-Paraná | Breeding Lines | Red - (Colors) | Mesoamerican | G2 |
| Line34 | IDR-Paraná | Breeding Lines | Pinto - (Colors) | Mesoamerican | G2 |
| Line35 | IDR-Paraná | Breeding Lines | Red - (Colors) | Mesoamerican | G2 |
| Line36 | IDR-Paraná | Breeding Lines | Pinto - (Colors) | Mesoamerican | G2 |
| Line37 | IDR-Paraná | Breeding Lines | Red - (Colors) | Mesoamerican | G2 |
| Line38 | IDR-Paraná | Breeding Lines | Pinto - (Colors) | Mesoamerican | G2 |
| Line39 | IDR-Paraná | Breeding Lines | Red - (Colors) | Mesoamerican | G1 |
| Line40 | IDR-Paraná | Breeding Lines | Red - (Colors) | Mesoamerican | G2 |
| Line41 | IDR-Paraná | Breeding Lines | Red - (Colors) | Mesoamerican | G2 |
| Line42 | IDR-Paraná | Breeding Lines | Red - (Colors) | Mesoamerican | G2 |
| Line43 | IDR-Paraná | Breeding Lines | Red - (Colors) | Mesoamerican | G2 |
| Line44 | IDR-Paraná | Breeding Lines | Red - (Colors) | Mesoamerican | G3 |
| Line45 | IDR-Paraná | Breeding Lines | Brown - (Colors) | Mesoamerican | G2 |
| Line46 | IDR-Paraná | Breeding Lines | Brown - (Colors) | Mesoamerican | G2 |
| Line47 | IDR-Paraná | Breeding Lines | Pinto - (Colors) | Mesoamerican | G3 |
| Line48 | IDR-Paraná | Breeding Lines | White - (Colors) | Mesoamerican | G3 |
| Line49 | IDR-Paraná | Breeding Lines | Brown - (Colors) | Mesoamerican | G1 |
| Line50 | IDR-Paraná | Breeding Lines | Red - (Colors) | Mesoamerican | G2 |
| Line51 | IDR-Paraná | Breeding Lines | Brown - (Colors) | Mesoamerican | G1 |
| Line52 | IDR-Paraná | Breeding Lines | Brown - (Colors) | Mesoamerican | G3 |
| Line53 | IDR-Paraná | Breeding Lines | White - (Colors) | Mesoamerican | G3 |
| Line54 | IDR-Paraná | Breeding Lines | Brown - (Colors) | Mesoamerican | G3 |
| Line55 | IDR-Paraná | Breeding Lines | Brown - (Colors) | Mesoamerican | G3 |
| Line56 | IDR-Paraná | Breeding Lines | Brown - (Colors) | Mesoamerican | G2 |
| Line57 | IDR-Paraná | Breeding Lines | Brown - (Colors) | Mesoamerican | G3 |
| Line58 | IDR-Paraná | Breeding Lines | Brown - (Colors) | Mesoamerican | G2 |
| Line59 | IDR-Paraná | Breeding Lines | Brown - (Colors) | Mesoamerican | G5 |
| Line60 | IDR-Paraná | Breeding Lines | Brown - (Colors) | Mesoamerican | G2 |
| Line61 | IDR-Paraná | Breeding Lines | Brown - (Colors) | Mesoamerican | G3 |
| Line62 | IDR-Paraná | Breeding Lines | Brown - (Colors) | Mesoamerican | G2 |
| Line63 | IDR-Paraná | Breeding Lines | Brown - (Colors) | Mesoamerican | G2 |
| Line64 | IDR-Paraná | Breeding Lines | Brown - (Colors) | Mesoamerican | G2 |
| BAT1192 | CIAT | Breeding Lines | Brown - (Colors) | Mesoamerican | G2 |
| BAT1215 | CIAT | Breeding Lines | Brown - (Colors) | Mesoamerican | G4 |
| BAT1220 | CIAT | Breeding Lines | Brown - (Colors) | Mesoamerican | G1 |
| BAT1289 | CIAT | Breeding Lines | White - (Colors) | Mesoamerican | G2 |
| BAT40 | CIAT | Breeding Lines | Brown - (Colors) | Mesoamerican | G3 |
| BAT41 | CIAT | Breeding Lines | Brown - (Colors) | Mesoamerican | G1 |
| BAT451 | CIAT | Breeding Lines | Brown - (Colors) | Mesoamerican | G3 |
| BAT58 | CIAT | Breeding Lines | Brown - (Colors) | Mesoamerican | G3 |
| BAT614 | CIAT | Breeding Lines | Brown - (Colors) | Mesoamerican | G1 |
| BAT67 | CIAT | Breeding Lines | Pinto - (Colors) | Mesoamerican | G3 |
| BAT76 | CIAT | Breeding Lines | White - (Colors) | Mesoamerican | G3 |
| BAT85 | CIAT | Breeding Lines | Brown - (Colors) | Mesoamerican | G3 |
| BAT93 | CIAT | Breeding Lines | Brown - (Colors) | Mesoamerican | G2 |
| Black Hawk | - | Cultivars | Pinto - (Colors) | Mesoamerican | G2 |
| Bolão Preto | - | Cultivars | Red - (Colors) | Mesoamerican | G3 |
| Brasil343 | - | Breeding Lines | Pinto - (Colors) | Mesoamerican | G3 |
| BRS Campeiro | Embrapa | Cultivars | Pinto - (Colors) | Mesoamerican | G2 |
| BRS Pontal | Embrapa | Cultivars | Brown - (Colors) | Mesoamerican | G2 |
| BRS Requinte | Embrapa | Cultivars | Purple - (Colors) | Mesoamerican | G2 |
| BRS Supremo | Embrapa | Cultivars | Pink - (Colors) | Mesoamerican | G3 |
| BRS Valente | Embrapa | Cultivars | Brown - (Colors) | Mesoamerican | G2 |
| BRU14 | CIAT | Breeding Lines | Purple - (Colors) | Mesoamerican | G4 |
| BRU19 | CIAT | Breeding Lines | Pinto - (Colors) | Mesoamerican | G4 |
| BZ16987 | - | Breeding Lines | Red - (Colors) | Mesoamerican | G3 |
| BZ23172 | - | Breeding Lines | Yellow - (Colors) | Mesoamerican | G3 |
| Caisan | - | Cultivars | Purple - (Colors) | Andean | G1 |
| Campeão | - | Cultivars | Brown - (Colors) | Mesoamerican | G4 |
| Campeão1 | - | Cultivars | Purple - (Colors) | Mesoamerican | G4 |
| INT2 | Farmers | Landraces | Brown - (Colors) | Mesoamerican | G3 |
| Carioca | - | Landraces | White - (Colors) | Mesoamerican | G5 |
| Carioca1070 | - | Landraces | Red - (Colors) | Mesoamerican | G5 |
| Carioca60 | - | Landraces | Brown - (Colors) | Mesoamerican | G5 |
| INT3 | Farmers | Landraces | White - (Colors) | Mesoamerican | G2 |
| Carioca preto | - | Landraces | White - (Colors) | Mesoamerican | G5 |
| Carnaval | - | Landraces | White - (Colors) | Mesoamerican | G2 |
| Carnaval alongado | - | Landraces | Brown - (Colors) | Andean | G2 |
| Line65 | IDR-Paraná | Breeding Lines | Pinto - (Colors) | Mesoamerican | G2 |
| Cavalo1 | - | Landraces | Red - (Colors) | Mesoamerican | G2 |
| Line66 | IDR-Paraná | Breeding Lines | Brown - (Colors) | Mesoamerican | G3 |
| Line67 | IDR-Paraná | Breeding Lines | Red - (Colors) | Mesoamerican | G2 |
| Line68 | IDR-Paraná | Breeding Lines | Red - (Colors) | Mesoamerican | G1 |
| Line69 | IDR-Paraná | Breeding Lines | Brown - (Colors) | Mesoamerican | G2 |
| CNF261 | Embrapa | Breeding Lines | Brown - (Colors) | Mesoamerican | G2 |
| CNF8612 | Embrapa | Breeding Lines | Red - (Colors) | Mesoamerican | G3 |
| CNF8613 | Embrapa | Breeding Lines | Black Carioca - (Colors) | Mesoamerican | G2 |
| CNF864 | Embrapa | Breeding Lines | Black Carioca - (Colors) | Mesoamerican | G1 |
| CNF865 | Embrapa | Breeding Lines | Yellow - (Colors) | Mesoamerican | G2 |
| CNF868 | Embrapa | Breeding Lines | Pink - (Colors) | Mesoamerican | G5 |
| CNF869 | Embrapa | Breeding Lines | Brown - (Colors) | Mesoamerican | G5 |
| CNF8716 | Embrapa | Breeding Lines | Brown - (Colors) | Mesoamerican | G2 |
| CNF8718 | Embrapa | Breeding Lines | Brown - (Colors) | Mesoamerican | G5 |
| CNF8719 | Embrapa | Breeding Lines | Purple - (Colors) | Mesoamerican | G4 |
| CNF8721 | Embrapa | Breeding Lines | Cream - (Colors) | Mesoamerican | G4 |
| CNF874 | Embrapa | Breeding Lines | Brown - (Colors) | Mesoamerican | G4 |
| CNF876 | Embrapa | Breeding Lines | Brown - (Colors) | Andean | G2 |
| CNF877 | Embrapa | Breeding Lines | White - (Colors) | Mesoamerican | G4 |
| CNFP8078 | Embrapa | Breeding Lines | Red - (Colors) | Mesoamerican | G3 |
| CNFP8080 | Embrapa | Breeding Lines | Brown - (Colors) | Mesoamerican | G2 |
| CNFP8081 | Embrapa | Breeding Lines | Brown - (Colors) | Mesoamerican | G2 |
| CNFP8083 | Embrapa | Breeding Lines | Red - (Colors) | Mesoamerican | G3 |
| CNFP8085 | Embrapa | Breeding Lines | Brown - (Colors) | Mesoamerican | G3 |
| CNFP8087 | Embrapa | Breeding Lines | Purple - (Colors) | Mesoamerican | G3 |
| CNFP8088 | Embrapa | Breeding Lines | Brown - (Colors) | Mesoamerican | G2 |
| CNFP8091 | Embrapa | Breeding Lines | Brown - (Colors) | Mesoamerican | G2 |
| CNFP8093 | Embrapa | Breeding Lines | Brown - (Colors) | Mesoamerican | G2 |
| CNFP8094 | Embrapa | Breeding Lines | Red - (Colors) | Mesoamerican | G2 |
| CNFP8095 | Embrapa | Breeding Lines | Brown - (Colors) | Mesoamerican | G3 |
| CNFP8097 | Embrapa | Breeding Lines | Pinto - (Colors) | Mesoamerican | G2 |
| CNFP8098 | Embrapa | Breeding Lines | Brown - (Colors) | Mesoamerican | G2 |
| CNFP8099 | Embrapa | Breeding Lines | Pinto - (Colors) | Mesoamerican | G3 |
| CNFP8100 | Embrapa | Breeding Lines | Red - (Colors) | Mesoamerican | G2 |
| CNFP8101 | Embrapa | Breeding Lines | Pink - (Colors) | Mesoamerican | G3 |
| CNFP8103 | Embrapa | Breeding Lines | Purple - (Colors) | Mesoamerican | G3 |
| CNFP8104 | Embrapa | Breeding Lines | Brown - (Colors) | Mesoamerican | G3 |
| CNFP8105 | Embrapa | Breeding Lines | Pinto - (Colors) | Mesoamerican | G2 |
| CNFP8106 | Embrapa | Breeding Lines | Red - (Colors) | Mesoamerican | G2 |
| CNFP8108 | Embrapa | Breeding Lines | Yellow - (Colors) | Mesoamerican | G2 |
| Line70 | IDR-Paraná | Breeding Lines | White - (Colors) | Mesoamerican | G3 |
| Line71 | IDR-Paraná | Breeding Lines | Pinto - (Colors) | Mesoamerican | G2 |
| INT4 | Farmers | Landraces | Brown - (Colors) | Mesoamerican | G3 |
| Diamante Negro | Embrapa | Cultivars | Red - (Colors) | Mesoamerican | G3 |
| DOR191 | CIAT | Breeding Lines | Pinto - (Colors) | Mesoamerican | G2 |
| DOR202 | CIAT | Breeding Lines | Brown - (Colors) | Mesoamerican | G2 |
| DOR310 | CIAT | Breeding Lines | Red - (Colors) | Mesoamerican | G3 |
| DOR350 | CIAT | Breeding Lines | Black Carioca - (Colors) | Mesoamerican | G2 |
| DOR351 | CIAT | Breeding Lines | Red - (Colors) | Mesoamerican | G3 |
| DOR364 | CIAT | Breeding Lines | Red - (Colors) | Mesoamerican | G2 |
| DOR365 | CIAT | Breeding Lines | Brown - (Colors) | Mesoamerican | G5 |
| DOR368 | CIAT | Breeding Lines | Brown - (Colors) | Mesoamerican | G2 |
| DOR370 | CIAT | Breeding Lines | Brown - (Colors) | Mesoamerican | G2 |
| DOR371 | CIAT | Breeding Lines | Pink - (Colors) | Mesoamerican | G2 |
| DOR372 | CIAT | Breeding Lines | Pinto - (Colors) | Mesoamerican | G2 |
| DOR373 | CIAT | Breeding Lines | Pink - (Colors) | Mesoamerican | G2 |
| DOR374 | CIAT | Breeding Lines | Pink - (Colors) | Mesoamerican | G2 |
| DOR375 | CIAT | Breeding Lines | Yellow - (Colors) | Mesoamerican | G4 |
| DOR445 | CIAT | Breeding Lines | Brown - (Colors) | Mesoamerican | G4 |
| DOR446 | CIAT | Breeding Lines | Brown - (Colors) | Mesoamerican | G2 |
| DOR468 | CIAT | Breeding Lines | Pinto - (Colors) | Mesoamerican | G2 |
| DOR481 | CIAT | Breeding Lines | Pinto - (Colors) | Mesoamerican | G1 |
| DOR483 | CIAT | Breeding Lines | Pinto - (Colors) | Mesoamerican | G2 |
| DOR500 | CIAT | Breeding Lines | Brown - (Colors) | Mesoamerican | G2 |
| DOR609 | CIAT | Breeding Lines | Brown - (Colors) | Mesoamerican | G2 |
| INT5 | Farmers | Landraces | Brown - (Colors) | Mesoamerican | G4 |
| DRK10 | CIAT | Cultivars | Brown - (Colors) | Andean | G2 |
| DRK3 | CIAT | Cultivars | Brown - (Colors) | Mesoamerican | G2 |
| Line72 | IDR-Paraná | Breeding Lines | Brown - (Colors) | Mesoamerican | G3 |
| EMP110 | CIAT | Breeding Lines | Brown - (Colors) | Mesoamerican | G3 |
| EMP178 | CIAT | Breeding Lines | Brown - (Colors) | Andean | G2 |
| EMP187 | CIAT | Breeding Lines | Brown - (Colors) | Mesoamerican | G3 |
| EMP189 | CIAT | Breeding Lines | Brown - (Colors) | Mesoamerican | G2 |
| EMP194 | CIAT | Breeding Lines | Pinto - (Colors) | Mesoamerican | G2 |
| EMP198 | CIAT | Breeding Lines | Pink - (Colors) | Mesoamerican | G2 |
| EMP250 | CIAT | Breeding Lines | Brown - (Colors) | Mesoamerican | G4 |
| EMP269 | CIAT | Breeding Lines | Brown - (Colors) | Mesoamerican | G2 |
| EMP385 | CIAT | Breeding Lines | Black Carioca - (Colors) | Mesoamerican | G2 |
| EMP403 | CIAT | Breeding Lines | Black Carioca - (Colors) | Mesoamerican | G2 |
| EMP404 | CIAT | Breeding Lines | Pinto - (Colors) | Mesoamerican | G3 |
| EMP405 | CIAT | Breeding Lines | Black Carioca - (Colors) | Mesoamerican | G4 |
| EMP407 | CIAT | Breeding Lines | Cream - (Colors) | Mesoamerican | G5 |
| EMP414 | CIAT | Breeding Lines | Pinto - (Colors) | Mesoamerican | G5 |
| EMP440 | CIAT | Breeding Lines | Yellow - (Colors) | Mesoamerican | G5 |
| EMP89 | CIAT | Breeding Lines | Brown - (Colors) | Mesoamerican | G3 |
| Engopa Ouro | - | Cultivars | Black Carioca - (Colors) | Mesoamerican | G2 |
| EMP173 | CIAT | Breeding Lines | Pinto - (Colors) | Mesoamerican | G1 |
| ESAL501 | ESALQ | Breeding Lines | Pinto - (Colors) | Mesoamerican | G2 |
| ESAL506 | ESALQ | Breeding Lines | Brown - (Colors) | Mesoamerican | G3 |
| ESAL508 | ESALQ | Breeding Lines | Brown - (Colors) | Mesoamerican | G2 |
| ESAL511 | ESALQ | Breeding Lines | Pinto - (Colors) | Mesoamerican | G2 |
| ESAL522 | ESALQ | Breeding Lines | Brown - (Colors) | Mesoamerican | G5 |
| ESAL531 | ESALQ | Breeding Lines | Pink - (Colors) | Mesoamerican | G5 |
| ESAL536 | ESALQ | Breeding Lines | Cream - (Colors) | Mesoamerican | G2 |
| ESAL579 | ESALQ | Breeding Lines | Black and White - (Colors) | Mesoamerican | G2 |
| ESAL580 | ESALQ | Breeding Lines | Brown - (Colors) | Mesoamerican | G2 |
| ESAL583 | ESALQ | Breeding Lines | Pinto - (Colors) | Mesoamerican | G5 |
| ESAL586 | ESALQ | Breeding Lines | White - (Colors) | Mesoamerican | G5 |
| ESAL633 | ESALQ | Breeding Lines | Brown - (Colors) | Mesoamerican | G5 |
| BRS Expedito | Embrapa | Cultivars | Brown - (Colors) | Mesoamerican | G2 |
| FEB204 | CIAT | Breeding Lines | Brown - (Colors) | Mesoamerican | G5 |
| FEB122 | CIAT | Breeding Lines | Brown - (Colors) | Mesoamerican | G2 |
| FEB127 | CIAT | Breeding Lines | Brown - (Colors) | Mesoamerican | G3 |
| FEB129 | CIAT | Breeding Lines | Brown - (Colors) | Mesoamerican | G5 |
| FEB137 | CIAT | Breeding Lines | Brown - (Colors) | Mesoamerican | G5 |
| FEB146 | CIAT | Breeding Lines | Brown - (Colors) | Mesoamerican | G3 |
| FEB149 | CIAT | Breeding Lines | Brown - (Colors) | Mesoamerican | G3 |
| FEB151 | CIAT | Breeding Lines | Brown - (Colors) | Mesoamerican | G3 |
| FEB154 | CIAT | Breeding Lines | Brown - (Colors) | Mesoamerican | G3 |
| FEB156 | CIAT | Breeding Lines | Brown - (Colors) | Mesoamerican | G4 |
| FEB159 | CIAT | Breeding Lines | Brown - (Colors) | Mesoamerican | G3 |
| FEB164 | CIAT | Breeding Lines | Brown - (Colors) | Mesoamerican | G2 |
| FEB178 | CIAT | Breeding Lines | Brown - (Colors) | Mesoamerican | G5 |
| FEB179 | CIAT | Breeding Lines | Brown - (Colors) | Mesoamerican | G5 |
| FEB180 | CIAT | Breeding Lines | Brown - (Colors) | Mesoamerican | G2 |
| FEB181 | CIAT | Breeding Lines | Brown - (Colors) | Mesoamerican | G5 |
| FEB182 | CIAT | Breeding Lines | Brown - (Colors) | Mesoamerican | G5 |
| FEB183 | CIAT | Breeding Lines | Brown - (Colors) | Mesoamerican | G5 |
| FEB186 | CIAT | Breeding Lines | Brown - (Colors) | Mesoamerican | G2 |
| FEB187 | CIAT | Breeding Lines | Brown - (Colors) | Mesoamerican | G3 |
| FEB188 | CIAT | Breeding Lines | Brown - (Colors) | Mesoamerican | G1 |
| FEB189 | CIAT | Breeding Lines | Brown - (Colors) | Mesoamerican | G5 |
| FEB190 | CIAT | Breeding Lines | Brown - (Colors) | Mesoamerican | G3 |
| FEB191 | CIAT | Breeding Lines | Brown - (Colors) | Mesoamerican | G2 |
| FEB192 | CIAT | Breeding Lines | Brown - (Colors) | Mesoamerican | G2 |
| FEB193 | CIAT | Breeding Lines | Brown - (Colors) | Mesoamerican | G2 |
| FEB194 | CIAT | Breeding Lines | Brown - (Colors) | Mesoamerican | G2 |
| FEB195 | CIAT | Breeding Lines | Brown - (Colors) | Mesoamerican | G4 |
| FEB196 | CIAT | Breeding Lines | Brown - (Colors) | Mesoamerican | G4 |
| FEB197 | CIAT | Breeding Lines | Brown - (Colors) | Mesoamerican | G3 |
| FEB198 | CIAT | Breeding Lines | Purple - (Colors) | Mesoamerican | G3 |
| FEB200 | CIAT | Breeding Lines | Brown - (Colors) | Mesoamerican | G5 |
| FEB36 | CIAT | Breeding Lines | Brown - (Colors) | Mesoamerican | G2 |
| FEB70 | CIAT | Breeding Lines | Brown - (Colors) | Mesoamerican | G3 |
| FEB75 | CIAT | Breeding Lines | Brown - (Colors) | Mesoamerican | G3 |
| Feijão Cavalo | Farmers | Landraces | Brown - (Colors) | Andean | G2 |
| FT 120 | FT Sementes | Cultivars | Brown - (Colors) | Mesoamerican | G2 |
| FT83120 | FT Sementes | Breeding Lines | Brown - (Colors) | Mesoamerican | G2 |
| FT841654 | FT Sementes | Breeding Lines | Brown - (Colors) | Mesoamerican | G2 |
| FT84293A | FT Sementes | Breeding Lines | Brown - (Colors) | Mesoamerican | G5 |
| FT84349 | FT Sementes | Breeding Lines | Brown - (Colors) | Mesoamerican | G4 |
| FT84398 | FT Sementes | Breeding Lines | Brown - (Colors) | Mesoamerican | G2 |
| FT85206 | FT Sementes | Breeding Lines | Brown - (Colors) | Mesoamerican | G2 |
| FT8563 | FT Sementes | Breeding Lines | Brown - (Colors) | Mesoamerican | G2 |
| FT Bonito | FT Sementes | Cultivars | Brown - (Colors) | Mesoamerican | G4 |
| FT Nobre | FT Sementes | Cultivars | Brown - (Colors) | Mesoamerican | G2 |
| FT Tarumã | FT Sementes | Cultivars | Brown - (Colors) | Mesoamerican | G2 |
| G03568 | CIAT | Breeding Lines | Brown - (Colors) | Mesoamerican | G3 |
| G11035 | CIAT | Breeding Lines | Brown - (Colors) | Mesoamerican | G2 |
| G11164 | CIAT | Breeding Lines | Brown - (Colors) | Andean | G3 |
| G11270 | CIAT | Breeding Lines | Brown - (Colors) | Mesoamerican | G3 |
| G11495 | CIAT | Breeding Lines | Brown - (Colors) | Mesoamerican | G2 |
| G11564 | CIAT | Breeding Lines | Brown - (Colors) | Mesoamerican | G1 |
| G11721 | CIAT | Breeding Lines | Brown - (Colors) | Mesoamerican | G2 |
| G1205 | CIAT | Breeding Lines | Black Carioca - (Colors) | Mesoamerican | G2 |
| G12168 | CIAT | Breeding Lines | Black Carioca - (Colors) | Mesoamerican | G3 |
| G122 | CIAT | Breeding Lines | Black Carioca - (Colors) | Andean | G1 |
| G12556 | CIAT | Breeding Lines | Black Carioca - (Colors) | Mesoamerican | G4 |
| G1261 | CIAT | Breeding Lines | Black Carioca - (Colors) | Mesoamerican | G1 |
| G1264 | CIAT | Breeding Lines | Brown - (Colors) | Mesoamerican | G3 |
| G1265 | CIAT | Breeding Lines | Black Carioca - (Colors) | Mesoamerican | G2 |
| G13571 | CIAT | Breeding Lines | Black Carioca - (Colors) | Mesoamerican | G1 |
| G13578 | CIAT | Breeding Lines | Brown - (Colors) | Mesoamerican | G2 |
| G13728 | CIAT | Breeding Lines | Black Carioca - (Colors) | Mesoamerican | G2 |
| G13778 | CIAT | Breeding Lines | Black Carioca - (Colors) | Andean | G2 |
| G13920 | CIAT | Breeding Lines | Brown - (Colors) | Mesoamerican | G2 |
| G14027 | CIAT | Breeding Lines | Brown - (Colors) | Mesoamerican | G1 |
| G1414 | CIAT | Breeding Lines | Brown - (Colors) | Mesoamerican | G1 |
| G1420 | CIAT | Breeding Lines | Brown - (Colors) | Andean | G1 |
| G14380 | CIAT | Breeding Lines | Brown - (Colors) | Mesoamerican | G3 |
| G1457 | CIAT | Breeding Lines | Brown - (Colors) | Mesoamerican | G2 |
| G14645 | CIAT | Breeding Lines | Brown - (Colors) | Mesoamerican | G1 |
| G148 | CIAT | Breeding Lines | Brown - (Colors) | Mesoamerican | G4 |
| G14866 | CIAT | Breeding Lines | Brown - (Colors) | Mesoamerican | G2 |
| G15112 | CIAT | Breeding Lines | Brown - (Colors) | Andean | G1 |
| G15137 | CIAT | Breeding Lines | Brown - (Colors) | Andean | G2 |
| G15337 | CIAT | Breeding Lines | Brown - (Colors) | Mesoamerican | G1 |
| G1688 | CIAT | Breeding Lines | Brown - (Colors) | Mesoamerican | G2 |
| G17085 | CIAT | Breeding Lines | Yellow - (Colors) | Mesoamerican | G2 |
| G17426 | CIAT | Breeding Lines | Black Carioca - (Colors) | Mesoamerican | G2 |
| G17427 | CIAT | Breeding Lines | Brown - (Colors) | Mesoamerican | G3 |
| G17666 | CIAT | Breeding Lines | Pink - (Colors) | Mesoamerican | G2 |
| G17722 | CIAT | Breeding Lines | Brown - (Colors) | Mesoamerican | G4 |
| G18134 | CIAT | Breeding Lines | Brown - (Colors) | Mesoamerican | G4 |
| G18141 | CIAT | Breeding Lines | Brown - (Colors) | Mesoamerican | G2 |
| G18244 | CIAT | Breeding Lines | Brown - (Colors) | Mesoamerican | G4 |
| G18249 | CIAT | Breeding Lines | Red - (Colors) | Mesoamerican | G4 |
| G18454 | CIAT | Breeding Lines | White - (Colors) | Mesoamerican | G4 |
| G18479 | CIAT | Breeding Lines | Red - (Colors) | Mesoamerican | G2 |
| G18980 | CIAT | Breeding Lines | Red - (Colors) | Mesoamerican | G4 |
| G190028 | CIAT | Breeding Lines | Red - (Colors) | Andean | G1 |
| G1920 | CIAT | Breeding Lines | Red - (Colors) | Mesoamerican | G4 |
| G19842 | CIAT | Breeding Lines | Red - (Colors) | Mesoamerican | G2 |
| G20854 | CIAT | Breeding Lines | Red - (Colors) | Andean | G2 |
| G20864 | CIAT | Breeding Lines | Red - (Colors) | Mesoamerican | G1 |
| G2093 | CIAT | Breeding Lines | Red - (Colors) | Mesoamerican | G1 |
| G21147 | CIAT | Breeding Lines | Red - (Colors) | Mesoamerican | G2 |
| G21714 | CIAT | Breeding Lines | Red - (Colors) | Mesoamerican | G2 |
| G21725 | CIAT | Breeding Lines | White - (Colors) | Mesoamerican | G2 |
| G22502 | CIAT | Breeding Lines | White - (Colors) | Andean | G1 |
| G22505 | CIAT | Breeding Lines | White - (Colors) | Mesoamerican | G1 |
| G22534 | CIAT | Breeding Lines | White - (Colors) | Mesoamerican | G4 |
| G23315 | CIAT | Breeding Lines | Red - (Colors) | Mesoamerican | G2 |
| G2358 | CIAT | Breeding Lines | Pinto - (Colors) | Mesoamerican | G4 |
| G2446 | CIAT | Breeding Lines | Brown - (Colors) | Mesoamerican | G3 |
| G2455 | CIAT | Breeding Lines | Brown - (Colors) | Mesoamerican | G4 |
| G2472 | CIAT | Breeding Lines | Brown - (Colors) | Mesoamerican | G2 |
| G2676 | CIAT | Breeding Lines | Red - (Colors) | Mesoamerican | G3 |
| G2681 | CIAT | Breeding Lines | Brown - (Colors) | Mesoamerican | G4 |
| G2868 | CIAT | Breeding Lines | Purple - (Colors) | Mesoamerican | G2 |
| G3005 | CIAT | Breeding Lines | Brown - (Colors) | Mesoamerican | G1 |
| G3178 | CIAT | Breeding Lines | Purple - (Colors) | Mesoamerican | G4 |
| G3217 | CIAT | Breeding Lines | Brown - (Colors) | Mesoamerican | G2 |
| G3512 | CIAT | Breeding Lines | Brown - (Colors) | Mesoamerican | G2 |
| G3530 | CIAT | Breeding Lines | Brown - (Colors) | Mesoamerican | G3 |
| G3566 | CIAT | Breeding Lines | Brown - (Colors) | Mesoamerican | G2 |
| G3593 | CIAT | Breeding Lines | Brown - (Colors) | Mesoamerican | G1 |
| G3645 | CIAT | Breeding Lines | Brown - (Colors) | Mesoamerican | G2 |
| G3646 | CIAT | Breeding Lines | Brown - (Colors) | Mesoamerican | G2 |
| G3807 | CIAT | Breeding Lines | Brown - (Colors) | Mesoamerican | G2 |
| G3971 | CIAT | Breeding Lines | White - (Colors) | Mesoamerican | G3 |
| G3990 | CIAT | Breeding Lines | Black Carioca - (Colors) | Mesoamerican | G4 |
| G4001 | CIAT | Breeding Lines | Black Carioca - (Colors) | Andean | G2 |
| G4030 | CIAT | Breeding Lines | Brown - (Colors) | Mesoamerican | G4 |
| G4338 | CIAT | Breeding Lines | Brown - (Colors) | Mesoamerican | G2 |
| G4399 | CIAT | Breeding Lines | Brown - (Colors) | Mesoamerican | G2 |
| G4462 | CIAT | Breeding Lines | Brown - (Colors) | Mesoamerican | G3 |
| G4489 | CIAT | Breeding Lines | Brown - (Colors) | Mesoamerican | G3 |
| G4644 | CIAT | Breeding Lines | Brown - (Colors) | Mesoamerican | G2 |
| G4769 | CIAT | Breeding Lines | Brown - (Colors) | Mesoamerican | G2 |
| G4790 | CIAT | Breeding Lines | Brown - (Colors) | Mesoamerican | G2 |
| G4791 | CIAT | Breeding Lines | Brown - (Colors) | Mesoamerican | G4 |
| G4822 | CIAT | Breeding Lines | Brown - (Colors) | Mesoamerican | G1 |
| G4825 | CIAT | Breeding Lines | Brown - (Colors) | Mesoamerican | G5 |
| G4830 | CIAT | Breeding Lines | Brown - (Colors) | Mesoamerican | G3 |
| G4837 | CIAT | Breeding Lines | Brown - (Colors) | Mesoamerican | G1 |
| G4970 | CIAT | Breeding Lines | Brown - (Colors) | Mesoamerican | G1 |
| G5129 | CIAT | Breeding Lines | Brown - (Colors) | Andean | G2 |
| G5201 | CIAT | Breeding Lines | Brown - (Colors) | Mesoamerican | G3 |
| G5266 | CIAT | Breeding Lines | Brown - (Colors) | Mesoamerican | G3 |
| G5285 | CIAT | Breeding Lines | Brown - (Colors) | Mesoamerican | G4 |
| G5433 | CIAT | Breeding Lines | Brown - (Colors) | Mesoamerican | G1 |
| G5439 | CIAT | Breeding Lines | Brown - (Colors) | Mesoamerican | G2 |
| G5652 | CIAT | Breeding Lines | Brown - (Colors) | Mesoamerican | G3 |
| G5706 | CIAT | Breeding Lines | Brown - (Colors) | Mesoamerican | G2 |
| G5725 | CIAT | Breeding Lines | Black Carioca - (Colors) | Mesoamerican | G2 |
| G5733 | CIAT | Breeding Lines | Black Carioca - (Colors) | Mesoamerican | G4 |
| G5773 | CIAT | Breeding Lines | Brown - (Colors) | Mesoamerican | G2 |
| G5902 | CIAT | Breeding Lines | Purple - (Colors) | Mesoamerican | G2 |
| G6115 | CIAT | Breeding Lines | Brown - (Colors) | Mesoamerican | G2 |
| G6450 | CIAT | Breeding Lines | Brown - (Colors) | Mesoamerican | G2 |
| G6762 | CIAT | Breeding Lines | Red - (Colors) | Mesoamerican | G1 |
| G734 | CIAT | Breeding Lines | White - (Colors) | Mesoamerican | G2 |
| G7474 | CIAT | Breeding Lines | White - (Colors) | Mesoamerican | G3 |
| G7932 | CIAT | Breeding Lines | White - (Colors) | Mesoamerican | G4 |
| G8086 | CIAT | Breeding Lines | White - (Colors) | Andean | G2 |
| G87 | CIAT | Breeding Lines | Pink - (Colors) | Mesoamerican | G4 |
| G8965 | CIAT | Breeding Lines | Pink - (Colors) | Mesoamerican | G4 |
| G92 | CIAT | Breeding Lines | Pink - (Colors) | Mesoamerican | G3 |
| G9263 | CIAT | Breeding Lines | Purple - (Colors) | Mesoamerican | G1 |
| G9553 | CIAT | Breeding Lines | Purple - (Colors) | Andean | G2 |
| G9899 | CIAT | Breeding Lines | Brown - (Colors) | Mesoamerican | G2 |
| Garça Carioca | Farmers | Landraces | Purple - (Colors) | Mesoamerican | G5 |
| GEN96A/45 | - | Breeding Lines | Purple - (Colors) | Mesoamerican | G4 |
| GEN96A/98 | - | Breeding Lines | Purple - (Colors) | Mesoamerican | G2 |
| GENC122 | - | Breeding Lines | Pink - (Colors) | Mesoamerican | G5 |
| GENC146 | - | Breeding Lines | Pinto - (Colors) | Mesoamerican | G5 |
| GENC971 | - | Breeding Lines | Red - (Colors) | Mesoamerican | G5 |
| GENC9710 | - | Breeding Lines | Red - (Colors) | Mesoamerican | G5 |
| GENC9714 | - | Breeding Lines | White - (Colors) | Mesoamerican | G5 |
| GENC9715 | - | Breeding Lines | White - (Colors) | Mesoamerican | G5 |
| GENC9717 | - | Breeding Lines | White - (Colors) | Mesoamerican | G5 |
| GENC9718 | - | Breeding Lines | White - (Colors) | Mesoamerican | G5 |
| GENC972 | - | Breeding Lines | Red - (Colors) | Mesoamerican | G5 |
| GENC977 | - | Breeding Lines | White - (Colors) | Mesoamerican | G5 |
| GENC978 | - | Breeding Lines | Red - (Colors) | Mesoamerican | G5 |
| GENC983 | - | Breeding Lines | Red - (Colors) | Mesoamerican | G5 |
| GX97922462 | - | Breeding Lines | Red - (Colors) | Mesoamerican | G5 |
| GX97922871 | - | Breeding Lines | Brown - (Colors) | Mesoamerican | G5 |
| GX97924905 | - | Breeding Lines | Pinto - (Colors) | Mesoamerican | G5 |
| GX97924984 | - | Breeding Lines | Carioca | Mesoamerican | G5 |
| GX97925142 | - | Breeding Lines | Carioca | Mesoamerican | G5 |
| GX97925332 | - | Breeding Lines | Carioca | Mesoamerican | G5 |
| GX97925955 | - | Breeding Lines | Carioca | Mesoamerican | G5 |
| GX97926072 | - | Breeding Lines | Carioca | Mesoamerican | G5 |
| Line73 | IDR-Paraná | Breeding Lines | Brown | Mesoamerican | G4 |
| Line74 | IDR-Paraná | Breeding Lines | Carioca | Mesoamerican | G2 |
| Line75 | IDR-Paraná | Breeding Lines | Brown | Mesoamerican | G2 |
| IAC1 | IAC | Cultivars | Pink | Mesoamerican | G1 |
| IAC Tybatã | IAC | Cultivars | Carioca | Mesoamerican | G5 |
| IAC Una | IAC | Cultivars | Black | Mesoamerican | G2 |
| IAPAR 14 | IDR-Paraná | Cultivars | Carioca | Mesoamerican | G3 |
| IAPAR 20 | IDR-Paraná | Cultivars | Black | Mesoamerican | G2 |
| IAPAR 31 | IDR-Paraná | Cultivars | Pinto | Mesoamerican | G1 |
| IAPAR 72 | IDR-Paraná | Cultivars | Carioca | Mesoamerican | G2 |
| IAPAR 80 | IDR-Paraná | Cultivars | Carioca | Mesoamerican | G2 |
| IAPAR 81 | IDR-Paraná | Cultivars | Carioca | Mesoamerican | G2 |
| IAPAR 811 | IDR-Paraná | Cultivars | Carioca | Mesoamerican | G5 |
| Line76 | IDR-Paraná | Breeding Lines | Pink | Mesoamerican | G1 |
| Line77 | IDR-Paraná | Breeding Lines | Pink | Mesoamerican | G1 |
| Line78 | IDR-Paraná | Breeding Lines | Yellow | Mesoamerican | G1 |
| Line79 | IDR-Paraná | Breeding Lines | Brown | Mesoamerican | G1 |
| Line80 | IDR-Paraná | Breeding Lines | Carioca | Mesoamerican | G2 |
| Line81 | IDR-Paraná | Breeding Lines | Carioca | Mesoamerican | G5 |
| Line82 | IDR-Paraná | Breeding Lines | Brown | Mesoamerican | G3 |
| Line83 | IDR-Paraná | Breeding Lines | Pinto | Mesoamerican | G1 |
| Line84 | IDR-Paraná | Breeding Lines | Pinto | Mesoamerican | G1 |
| Line85 | IDR-Paraná | Breeding Lines | Pinto | Mesoamerican | G2 |
| Line86 | IDR-Paraná | Breeding Lines | Brown | Mesoamerican | G2 |
| Line87 | IDR-Paraná | Breeding Lines | Carioca | Mesoamerican | G5 |
| Line88 | IDR-Paraná | Breeding Lines | Black | Mesoamerican | G2 |
| Line89 | IDR-Paraná | Breeding Lines | Black | Mesoamerican | G2 |
| Line90 | IDR-Paraná | Breeding Lines | Black | Mesoamerican | G2 |
| Line91 | IDR-Paraná | Breeding Lines | Black | Mesoamerican | G2 |
| Line92 | IDR-Paraná | Breeding Lines | Brown | Mesoamerican | G2 |
| Line93 | IDR-Paraná | Breeding Lines | Brown | Mesoamerican | G3 |
| Line94 | IDR-Paraná | Breeding Lines | Black | Mesoamerican | G3 |
| Line95 | IDR-Paraná | Breeding Lines | Black | Mesoamerican | G3 |
| Line96 | IDR-Paraná | Breeding Lines | Black | Mesoamerican | G2 |
| Line97 | IDR-Paraná | Breeding Lines | Black | Mesoamerican | G2 |
| Line98 | IDR-Paraná | Breeding Lines | Black | Mesoamerican | G2 |
| Line99 | IDR-Paraná | Breeding Lines | Black | Mesoamerican | G2 |
| Line100 | IDR-Paraná | Breeding Lines | Black | Mesoamerican | G3 |
| Line101 | IDR-Paraná | Breeding Lines | Black | Mesoamerican | G3 |
| Line102 | IDR-Paraná | Breeding Lines | Brown | Mesoamerican | G2 |
| Line103 | IDR-Paraná | Breeding Lines | Black | Mesoamerican | G2 |
| Line104 | IDR-Paraná | Breeding Lines | Black | Mesoamerican | G3 |
| Line105 | IDR-Paraná | Breeding Lines | Black | Mesoamerican | G3 |
| Line106 | IDR-Paraná | Breeding Lines | Black | Mesoamerican | G3 |
| Line107 | IDR-Paraná | Breeding Lines | Black | Mesoamerican | G2 |
| Line108 | IDR-Paraná | Breeding Lines | Brown | Mesoamerican | G2 |
| Line109 | IDR-Paraná | Breeding Lines | Black | Mesoamerican | G3 |
| Line110 | IDR-Paraná | Breeding Lines | Black | Mesoamerican | G2 |
| Line111 | IDR-Paraná | Breeding Lines | Black | Mesoamerican | G2 |
| Line112 | IDR-Paraná | Breeding Lines | Carioca | Mesoamerican | G5 |
| Line113 | IDR-Paraná | Breeding Lines | Carioca | Mesoamerican | G5 |
| Line114 | IDR-Paraná | Breeding Lines | Carioca | Mesoamerican | G5 |
| Line115 | IDR-Paraná | Breeding Lines | Brown | Mesoamerican | G3 |
| Line116 | IDR-Paraná | Breeding Lines | Brown | Mesoamerican | G2 |
| Line117 | IDR-Paraná | Breeding Lines | Brown | Mesoamerican | G3 |
| Line118 | IDR-Paraná | Breeding Lines | Brown | Mesoamerican | G2 |
| Line119 | IDR-Paraná | Breeding Lines | Black | Mesoamerican | G2 |
| Line120 | IDR-Paraná | Breeding Lines | Carioca | Mesoamerican | G5 |
| Line121 | IDR-Paraná | Breeding Lines | Brown | Mesoamerican | G2 |
| Line122 | IDR-Paraná | Breeding Lines | Pinto | Mesoamerican | G1 |
| Line123 | IDR-Paraná | Breeding Lines | Carioca | Mesoamerican | G2 |
| Line124 | IDR-Paraná | Breeding Lines | Pink | Mesoamerican | G1 |
| Line125 | IDR-Paraná | Breeding Lines | Brown | Mesoamerican | G2 |
| Line126 | IDR-Paraná | Breeding Lines | Carioca | Mesoamerican | G2 |
| Line127 | IDR-Paraná | Breeding Lines | Carioca | Mesoamerican | G5 |
| Line128 | IDR-Paraná | Breeding Lines | Brown | Mesoamerican | G3 |
| Line129 | IDR-Paraná | Breeding Lines | Carioca | Mesoamerican | G3 |
| Line130 | IDR-Paraná | Breeding Lines | Black Carioca | Mesoamerican | G2 |
| Line131 | IDR-Paraná | Breeding Lines | Black | Mesoamerican | G3 |
| Line132 | IDR-Paraná | Breeding Lines | Black | Mesoamerican | G2 |
| Line133 | IDR-Paraná | Breeding Lines | Black | Mesoamerican | G2 |
| Line134 | IDR-Paraná | Breeding Lines | Black Carioca | Mesoamerican | G2 |
| Line135 | IDR-Paraná | Breeding Lines | Pinto | Mesoamerican | G2 |
| Line136 | IDR-Paraná | Breeding Lines | Black Carioca | Mesoamerican | G2 |
| Line137 | IDR-Paraná | Breeding Lines | Cream | Mesoamerican | G2 |
| Line138 | IDR-Paraná | Breeding Lines | Pinto | Mesoamerican | G2 |
| Line139 | IDR-Paraná | Breeding Lines | Yellow | Mesoamerican | G2 |
| Line140 | IDR-Paraná | Breeding Lines | Black | Mesoamerican | G2 |
| Line141 | IDR-Paraná | Breeding Lines | Black | Mesoamerican | G2 |
| Line142 | IDR-Paraná | Breeding Lines | Brown | Mesoamerican | G2 |
| Line143 | IDR-Paraná | Breeding Lines | Black Carioca | Mesoamerican | G2 |
| Line144 | IDR-Paraná | Breeding Lines | Pinto | Mesoamerican | G2 |
| Line145 | IDR-Paraná | Breeding Lines | Pinto | Mesoamerican | G2 |
| Line146 | IDR-Paraná | Breeding Lines | Brown | Mesoamerican | G4 |
| Line147 | IDR-Paraná | Breeding Lines | Black | Mesoamerican | G2 |
| Line148 | IDR-Paraná | Breeding Lines | Brown | Mesoamerican | G2 |
| Line149 | IDR-Paraná | Breeding Lines | Pinto | Mesoamerican | G2 |
| Line150 | IDR-Paraná | Breeding Lines | Brown | Mesoamerican | G2 |
| Line151 | IDR-Paraná | Breeding Lines | Black | Mesoamerican | G2 |
| Line152 | IDR-Paraná | Breeding Lines | Pink | Mesoamerican | G1 |
| ICA Pijão | Instituto Colombiano Agropecuário | Cultivars | Black | Mesoamerican | G3 |
| ICA Quetzal | Instituto Colombiano Agropecuário | Cultivars | Black | Mesoamerican | G3 |
| ICA Tui | Instituto Colombiano Agropecuário | Cultivars | Black | Mesoamerican | G3 |
| INT6 | Farmers | Landraces | Carioca | Mesoamerican | G5 |
| INT7 | Farmers | Landraces | Carioca | Mesoamerican | G2 |
| INT8 | Farmers | Landraces | Carioca | Mesoamerican | G2 |
| INT9 | Farmers | Landraces | Carioca | Mesoamerican | G5 |
| INT10 | Farmers | Landraces | Carioca | Mesoamerican | G5 |
| INT11 | Farmers | Landraces | Carioca | Mesoamerican | G5 |
| INT12 | Farmers | Landraces | Carioca | Mesoamerican | G5 |
| INT13 | Farmers | Landraces | Cream | Andean | G1 |
| INT14 | Farmers | Landraces | Black and White | Andean | G1 |
| INT15 | Farmers | Landraces | Brown | Mesoamerican | G3 |
| IPR Chopim | IDR-Paraná | Cultivars | Black | Mesoamerican | G2 |
| IPR Colibri | IDR-Paraná | Cultivars | Carioca | Mesoamerican | G2 |
| IPR Corujinha | IDR-Paraná | Cultivars | Pinto | Mesoamerican | G2 |
| IPR Eldourado | IDR-Paraná | Cultivars | Carioca | Mesoamerican | G2 |
| IPR Garça | IDR-Paraná | Cultivars | White | Mesoamerican | G2 |
| IPR Gralha | IDR-Paraná | Cultivars | Black | Mesoamerican | G2 |
| IPR Graúna | IDR-Paraná | Cultivars | Black | Mesoamerican | G2 |
| IPR Juriti | IDR-Paraná | Cultivars | Carioca | Mesoamerican | G2 |
| IPR Saracura | IDR-Paraná | Cultivars | Carioca | Mesoamerican | G2 |
| IPR Siriri | IDR-Paraná | Cultivars | Carioca | Mesoamerican | G2 |
| IPR Tangara | IDR-Paraná | Cultivars | Carioca | Mesoamerican | G5 |
| IPR Tiziu | IDR-Paraná | Cultivars | Black | Mesoamerican | G2 |
| IPR Uirapuru | IDR-Paraná | Cultivars | Black | Mesoamerican | G2 |
| INT16 | Farmers | Landraces | Black | Mesoamerican | G3 |
| INT17 | Farmers | Landraces | Black | Mesoamerican | G2 |
| CNF243 | Embrapa | Breeding Lines | Brown | Andean | G1 |
| CNF260 | Embrapa | Breeding Lines | Brown | Andean | G1 |
| INT18 | Farmers | Landraces | Brown | Andean | G1 |
| JU898 | CIAT | Breeding Lines | Black | Mesoamerican | G3 |
| JW919 | CIAT | Breeding Lines | Black | Mesoamerican | G3 |
| Line153 | IDR-Paraná | Breeding Lines | Carioca | Mesoamerican | G5 |
| Line154 | IDR-Paraná | Breeding Lines | Black | Mesoamerican | G2 |
| Line155 | IDR-Paraná | Breeding Lines | Black | Mesoamerican | G4 |
| Line156 | IDR-Paraná | Breeding Lines | Carioca | Mesoamerican | G5 |
| Line157 | IDR-Paraná | Breeding Lines | Carioca | Mesoamerican | G5 |
| Line158 | IDR-Paraná | Breeding Lines | Black | Mesoamerican | G2 |
| Line159 | IDR-Paraná | Breeding Lines | Black | Mesoamerican | G2 |
| Line160 | IDR-Paraná | Breeding Lines | Black | Mesoamerican | G3 |
| Line161 | IDR-Paraná | Breeding Lines | Black | Mesoamerican | G3 |
| Line162 | IDR-Paraná | Breeding Lines | Black | Mesoamerican | G2 |
| Line163 | IDR-Paraná | Breeding Lines | Black | Mesoamerican | G2 |
| Line164 | IDR-Paraná | Breeding Lines | Black | Mesoamerican | G2 |
| Line165 | IDR-Paraná | Breeding Lines | Black | Mesoamerican | G2 |
| Line166 | IDR-Paraná | Breeding Lines | Carioca | Mesoamerican | G5 |
| Line167 | IDR-Paraná | Breeding Lines | Carioca | Mesoamerican | G5 |
| Line168 | IDR-Paraná | Breeding Lines | Carioca | Mesoamerican | G5 |
| Line169 | IDR-Paraná | Breeding Lines | Carioca | Mesoamerican | G5 |
| Line170 | IDR-Paraná | Breeding Lines | Carioca | Mesoamerican | G5 |
| Line171 | IDR-Paraná | Breeding Lines | Carioca | Mesoamerican | G5 |
| Line172 | IDR-Paraná | Breeding Lines | Black | Mesoamerican | G3 |
| Line173 | IDR-Paraná | Breeding Lines | Black | Mesoamerican | G2 |
| Line174 | IDR-Paraná | Breeding Lines | Carioca | Mesoamerican | G5 |
| Line175 | IDR-Paraná | Breeding Lines | Carioca | Mesoamerican | G5 |
| Line176 | IDR-Paraná | Breeding Lines | Carioca | Mesoamerican | G5 |
| Line177 | IDR-Paraná | Breeding Lines | Carioca | Mesoamerican | G5 |
| Line178 | IDR-Paraná | Breeding Lines | Carioca | Mesoamerican | G5 |
| Line179 | IDR-Paraná | Breeding Lines | Carioca | Mesoamerican | G5 |
| Line180 | IDR-Paraná | Breeding Lines | Carioca | Mesoamerican | G5 |
| Line181 | IDR-Paraná | Breeding Lines | Carioca | Mesoamerican | G5 |
| Line182 | IDR-Paraná | Breeding Lines | Carioca | Mesoamerican | G5 |
| Line183 | IDR-Paraná | Breeding Lines | Carioca | Mesoamerican | G5 |
| Line184 | IDR-Paraná | Breeding Lines | Carioca | Mesoamerican | G5 |
| Line185 | IDR-Paraná | Breeding Lines | Carioca | Mesoamerican | G5 |
| Line186 | IDR-Paraná | Breeding Lines | Carioca | Mesoamerican | G2 |
| Line187 | IDR-Paraná | Breeding Lines | Carioca | Mesoamerican | G2 |
| Line188 | IDR-Paraná | Breeding Lines | Carioca | Mesoamerican | G5 |
| Line189 | IDR-Paraná | Breeding Lines | Carioca | Mesoamerican | G5 |
| Line190 | IDR-Paraná | Breeding Lines | Carioca | Mesoamerican | G5 |
| Line191 | IDR-Paraná | Breeding Lines | Carioca | Mesoamerican | G2 |
| Line192 | IDR-Paraná | Breeding Lines | Black | Mesoamerican | G2 |
| Line193 | IDR-Paraná | Breeding Lines | Black | Mesoamerican | G2 |
| Line194 | IDR-Paraná | Breeding Lines | Black | Mesoamerican | G3 |
| Line195 | IDR-Paraná | Breeding Lines | Black | Mesoamerican | G2 |
| Line196 | IDR-Paraná | Breeding Lines | Black | Mesoamerican | G2 |
| Line197 | IDR-Paraná | Breeding Lines | Black | Mesoamerican | G2 |
| Line198 | IDR-Paraná | Breeding Lines | Black | Mesoamerican | G2 |
| Line199 | IDR-Paraná | Breeding Lines | Black | Mesoamerican | G3 |
| Line200 | IDR-Paraná | Breeding Lines | Black | Mesoamerican | G2 |
| Line201 | IDR-Paraná | Breeding Lines | Black | Mesoamerican | G2 |
| Line202 | IDR-Paraná | Breeding Lines | Black | Mesoamerican | G2 |
| Line203 | IDR-Paraná | Breeding Lines | Black | Mesoamerican | G2 |
| Line204 | IDR-Paraná | Breeding Lines | Black | Mesoamerican | G2 |
| Line205 | IDR-Paraná | Breeding Lines | Black | Mesoamerican | G2 |
| Line206 | IDR-Paraná | Breeding Lines | Black | Mesoamerican | G2 |
| Line207 | IDR-Paraná | Breeding Lines | Black | Mesoamerican | G2 |
| Line208 | IDR-Paraná | Breeding Lines | Carioca | Mesoamerican | G4 |
| Line209 | IDR-Paraná | Breeding Lines | Carioca | Mesoamerican | G2 |
| Line210 | IDR-Paraná | Breeding Lines | Carioca | Mesoamerican | G2 |
| Line211 | IDR-Paraná | Breeding Lines | Carioca | Mesoamerican | G2 |
| Line212 | IDR-Paraná | Breeding Lines | Carioca | Mesoamerican | G4 |
| Line213 | IDR-Paraná | Breeding Lines | Carioca | Mesoamerican | G4 |
| Line214 | IDR-Paraná | Breeding Lines | Carioca | Mesoamerican | G4 |
| Line215 | IDR-Paraná | Breeding Lines | Carioca | Mesoamerican | G4 |
| Line216 | IDR-Paraná | Breeding Lines | Carioca | Mesoamerican | G4 |
| Line217 | IDR-Paraná | Breeding Lines | Carioca | Mesoamerican | G2 |
| Line218 | IDR-Paraná | Breeding Lines | Carioca | Mesoamerican | G4 |
| Line219 | IDR-Paraná | Breeding Lines | Carioca | Mesoamerican | G4 |
| Line220 | IDR-Paraná | Breeding Lines | Carioca | Mesoamerican | G3 |
| Line221 | IDR-Paraná | Breeding Lines | Carioca | Mesoamerican | G4 |
| Line222 | IDR-Paraná | Breeding Lines | Carioca | Mesoamerican | G4 |
| Line223 | IDR-Paraná | Breeding Lines | Carioca | Mesoamerican | G4 |
| Line224 | IDR-Paraná | Breeding Lines | Carioca | Mesoamerican | G4 |
| Line225 | IDR-Paraná | Breeding Lines | Carioca | Mesoamerican | G2 |
| Line226 | IDR-Paraná | Breeding Lines | Carioca | Mesoamerican | G4 |
| Line227 | IDR-Paraná | Breeding Lines | Carioca | Mesoamerican | G4 |
| Line228 | IDR-Paraná | Breeding Lines | Carioca | Mesoamerican | G4 |
| Line229 | IDR-Paraná | Breeding Lines | Carioca | Mesoamerican | G4 |
| Line230 | IDR-Paraná | Breeding Lines | Carioca | Mesoamerican | G2 |
| Line231 | IDR-Paraná | Breeding Lines | Carioca | Mesoamerican | G4 |
| Line232 | IDR-Paraná | Breeding Lines | Carioca | Mesoamerican | G4 |
| Line233 | IDR-Paraná | Breeding Lines | Carioca | Mesoamerican | G4 |
| Line234 | IDR-Paraná | Breeding Lines | Carioca | Mesoamerican | G4 |
| Line235 | IDR-Paraná | Breeding Lines | Carioca | Mesoamerican | G4 |
| Line236 | IDR-Paraná | Breeding Lines | Carioca | Mesoamerican | G2 |
| Line237 | IDR-Paraná | Breeding Lines | Carioca | Mesoamerican | G4 |
| Line238 | IDR-Paraná | Breeding Lines | Carioca | Mesoamerican | G4 |
| Line239 | IDR-Paraná | Breeding Lines | Carioca | Mesoamerican | G4 |
| Line240 | IDR-Paraná | Breeding Lines | Carioca | Mesoamerican | G4 |
| Line241 | IDR-Paraná | Breeding Lines | Carioca | Mesoamerican | G4 |
| Line242 | IDR-Paraná | Breeding Lines | Carioca | Mesoamerican | G4 |
| Line243 | IDR-Paraná | Breeding Lines | Carioca | Mesoamerican | G4 |
| Line244 | IDR-Paraná | Breeding Lines | Carioca | Mesoamerican | G4 |
| Line245 | IDR-Paraná | Breeding Lines | Carioca | Mesoamerican | G4 |
| Line246 | IDR-Paraná | Breeding Lines | Carioca | Mesoamerican | G4 |
| Line247 | IDR-Paraná | Breeding Lines | Carioca | Mesoamerican | G4 |
| Line248 | IDR-Paraná | Breeding Lines | Carioca | Mesoamerican | G4 |
| Line249 | IDR-Paraná | Breeding Lines | Carioca | Mesoamerican | G4 |
| Line250 | IDR-Paraná | Breeding Lines | Carioca | Mesoamerican | G4 |
| Line251 | IDR-Paraná | Breeding Lines | Carioca | Mesoamerican | G4 |
| Line252 | IDR-Paraná | Breeding Lines | Carioca | Mesoamerican | G4 |
| Line253 | IDR-Paraná | Breeding Lines | Carioca | Mesoamerican | G4 |
| Line254 | IDR-Paraná | Breeding Lines | Carioca | Mesoamerican | G4 |
| Line255 | IDR-Paraná | Breeding Lines | Carioca | Mesoamerican | G4 |
| Line256 | IDR-Paraná | Breeding Lines | Carioca | Mesoamerican | G4 |
| Line257 | IDR-Paraná | Breeding Lines | Carioca | Mesoamerican | G4 |
| Line258 | IDR-Paraná | Breeding Lines | Carioca | Mesoamerican | G4 |
| Line259 | IDR-Paraná | Breeding Lines | Carioca | Mesoamerican | G4 |
| Line260 | IDR-Paraná | Breeding Lines | Black | Mesoamerican | G2 |
| Line261 | IDR-Paraná | Breeding Lines | Black | Mesoamerican | G2 |
| Line262 | IDR-Paraná | Breeding Lines | Black | Mesoamerican | G2 |
| Line263 | IDR-Paraná | Breeding Lines | Black | Mesoamerican | G2 |
| Line264 | IDR-Paraná | Breeding Lines | Black | Mesoamerican | G2 |
| Line265 | IDR-Paraná | Breeding Lines | Black | Mesoamerican | G2 |
| Line266 | IDR-Paraná | Breeding Lines | Black | Mesoamerican | G2 |
| Line267 | IDR-Paraná | Breeding Lines | Black | Mesoamerican | G2 |
| Line268 | IDR-Paraná | Breeding Lines | Black | Mesoamerican | G2 |
| Line269 | IDR-Paraná | Breeding Lines | Black | Mesoamerican | G2 |
| Line270 | IDR-Paraná | Breeding Lines | Black | Mesoamerican | G2 |
| Line271 | IDR-Paraná | Breeding Lines | Black | Mesoamerican | G3 |
| Line272 | IDR-Paraná | Breeding Lines | Black | Mesoamerican | G2 |
| Line273 | IDR-Paraná | Breeding Lines | Black | Mesoamerican | G2 |
| Line274 | IDR-Paraná | Breeding Lines | Black | Mesoamerican | G2 |
| Line275 | IDR-Paraná | Breeding Lines | Black | Mesoamerican | G2 |
| Line276 | IDR-Paraná | Breeding Lines | Black | Mesoamerican | G2 |
| Line277 | IDR-Paraná | Breeding Lines | Black | Mesoamerican | G2 |
| Line278 | IDR-Paraná | Breeding Lines | Black | Mesoamerican | G2 |
| Line279 | IDR-Paraná | Breeding Lines | Black | Mesoamerican | G2 |
| Line280 | IDR-Paraná | Breeding Lines | Black | Mesoamerican | G2 |
| Line281 | IDR-Paraná | Breeding Lines | Black | Mesoamerican | G2 |
| Line282 | IDR-Paraná | Breeding Lines | Black | Mesoamerican | G2 |
| Line283 | IDR-Paraná | Breeding Lines | Black | Mesoamerican | G2 |
| Line284 | IDR-Paraná | Breeding Lines | Black | Mesoamerican | G2 |
| Line285 | IDR-Paraná | Breeding Lines | Black | Mesoamerican | G2 |
| Line286 | IDR-Paraná | Breeding Lines | Black | Mesoamerican | G2 |
| Line287 | IDR-Paraná | Breeding Lines | Black | Mesoamerican | G2 |
| Line288 | IDR-Paraná | Breeding Lines | Black | Mesoamerican | G2 |
| Line289 | IDR-Paraná | Breeding Lines | Black | Mesoamerican | G2 |
| Line290 | IDR-Paraná | Breeding Lines | Black | Mesoamerican | G2 |
| Line291 | IDR-Paraná | Breeding Lines | Black | Mesoamerican | G2 |
| Line292 | IDR-Paraná | Breeding Lines | Black | Mesoamerican | G2 |
| Line293 | IDR-Paraná | Breeding Lines | Black | Mesoamerican | G2 |
| Line294 | IDR-Paraná | Breeding Lines | Black | Mesoamerican | G2 |
| Line295 | IDR-Paraná | Breeding Lines | Black | Mesoamerican | G2 |
| Line296 | IDR-Paraná | Breeding Lines | Black | Mesoamerican | G2 |
| Line297 | IDR-Paraná | Breeding Lines | Black | Mesoamerican | G2 |
| Line298 | IDR-Paraná | Breeding Lines | Black | Mesoamerican | G2 |
| Line299 | IDR-Paraná | Breeding Lines | Black | Mesoamerican | G2 |
| Line300 | IDR-Paraná | Breeding Lines | Black | Mesoamerican | G2 |
| Line301 | IDR-Paraná | Breeding Lines | Black | Mesoamerican | G2 |
| Line302 | IDR-Paraná | Breeding Lines | Black | Mesoamerican | G2 |
| Line303 | IDR-Paraná | Breeding Lines | Black | Mesoamerican | G2 |
| Line304 | IDR-Paraná | Breeding Lines | Black | Mesoamerican | G2 |
| Line305 | IDR-Paraná | Breeding Lines | Black | Mesoamerican | G2 |
| Line306 | IDR-Paraná | Breeding Lines | Black | Mesoamerican | G2 |
| Line307 | IDR-Paraná | Breeding Lines | Black | Mesoamerican | G2 |
| Line308 | IDR-Paraná | Breeding Lines | Black | Mesoamerican | G2 |
| Line309 | IDR-Paraná | Breeding Lines | Black | Mesoamerican | G2 |
| Line310 | IDR-Paraná | Breeding Lines | Carioca | Mesoamerican | G2 |
| Line311 | IDR-Paraná | Breeding Lines | Carioca | Mesoamerican | G2 |
| Line312 | IDR-Paraná | Breeding Lines | Carioca | Mesoamerican | G2 |
| Line313 | IDR-Paraná | Breeding Lines | Carioca | Mesoamerican | G2 |
| Line314 | IDR-Paraná | Breeding Lines | Carioca | Mesoamerican | G2 |
| Line315 | IDR-Paraná | Breeding Lines | Carioca | Mesoamerican | G4 |
| Line316 | IDR-Paraná | Breeding Lines | Carioca | Mesoamerican | G4 |
| Line317 | IDR-Paraná | Breeding Lines | Carioca | Mesoamerican | G4 |
| Line318 | IDR-Paraná | Breeding Lines | Carioca | Mesoamerican | G4 |
| Line319 | IDR-Paraná | Breeding Lines | Carioca | Mesoamerican | G4 |
| Line320 | IDR-Paraná | Breeding Lines | Carioca | Mesoamerican | G4 |
| Line321 | IDR-Paraná | Breeding Lines | Carioca | Mesoamerican | G4 |
| Line322 | IDR-Paraná | Breeding Lines | Carioca | Mesoamerican | G4 |
| Line323 | IDR-Paraná | Breeding Lines | Carioca | Mesoamerican | G4 |
| Line324 | IDR-Paraná | Breeding Lines | Carioca | Mesoamerican | G4 |
| Line325 | IDR-Paraná | Breeding Lines | Carioca | Mesoamerican | G4 |
| Line326 | IDR-Paraná | Breeding Lines | Carioca | Mesoamerican | G4 |
| Line327 | IDR-Paraná | Breeding Lines | Carioca | Mesoamerican | G4 |
| Line328 | IDR-Paraná | Breeding Lines | Carioca | Mesoamerican | G4 |
| Line329 | IDR-Paraná | Breeding Lines | Carioca | Mesoamerican | G4 |
| Line330 | IDR-Paraná | Breeding Lines | Carioca | Mesoamerican | G4 |
| Line331 | IDR-Paraná | Breeding Lines | Carioca | Mesoamerican | G4 |
| Line332 | IDR-Paraná | Breeding Lines | Carioca | Mesoamerican | G4 |
| Line333 | IDR-Paraná | Breeding Lines | Carioca | Mesoamerican | G4 |
| Line334 | IDR-Paraná | Breeding Lines | Carioca | Mesoamerican | G3 |
| Line335 | IDR-Paraná | Breeding Lines | Carioca | Mesoamerican | G4 |
| Line336 | IDR-Paraná | Breeding Lines | Carioca | Mesoamerican | G4 |
| Line337 | IDR-Paraná | Breeding Lines | Carioca | Mesoamerican | G4 |
| Line338 | IDR-Paraná | Breeding Lines | Carioca | Mesoamerican | G4 |
| Line339 | IDR-Paraná | Breeding Lines | Carioca | Mesoamerican | G4 |
| Line340 | IDR-Paraná | Breeding Lines | Carioca | Mesoamerican | G4 |
| Line341 | IDR-Paraná | Breeding Lines | Carioca | Mesoamerican | G4 |
| Line342 | IDR-Paraná | Breeding Lines | Carioca | Mesoamerican | G4 |
| Line343 | IDR-Paraná | Breeding Lines | Carioca | Mesoamerican | G4 |
| Line344 | IDR-Paraná | Breeding Lines | Carioca | Mesoamerican | G4 |
| Line345 | IDR-Paraná | Breeding Lines | Carioca | Mesoamerican | G4 |
| Line346 | IDR-Paraná | Breeding Lines | Carioca | Mesoamerican | G4 |
| Line347 | IDR-Paraná | Breeding Lines | Carioca | Mesoamerican | G2 |
| Line348 | IDR-Paraná | Breeding Lines | Carioca | Mesoamerican | G2 |
| Line349 | IDR-Paraná | Breeding Lines | Carioca | Mesoamerican | G2 |
| Line350 | IDR-Paraná | Breeding Lines | Carioca | Mesoamerican | G4 |
| Line351 | IDR-Paraná | Breeding Lines | Carioca | Mesoamerican | G4 |
| Line352 | IDR-Paraná | Breeding Lines | Carioca | Mesoamerican | G4 |
| Line353 | IDR-Paraná | Breeding Lines | Carioca | Mesoamerican | G4 |
| Line354 | IDR-Paraná | Breeding Lines | Carioca | Mesoamerican | G4 |
| Line355 | IDR-Paraná | Breeding Lines | Carioca | Mesoamerican | G4 |
| Line356 | IDR-Paraná | Breeding Lines | Carioca | Mesoamerican | G4 |
| Line357 | IDR-Paraná | Breeding Lines | Carioca | Mesoamerican | G4 |
| Line358 | IDR-Paraná | Breeding Lines | Carioca | Mesoamerican | G4 |
| Line359 | IDR-Paraná | Breeding Lines | Carioca | Mesoamerican | G4 |
| Line360 | IDR-Paraná | Breeding Lines | Carioca | Mesoamerican | G3 |
| Line361 | IDR-Paraná | Breeding Lines | Carioca | Mesoamerican | G2 |
| Line362 | IDR-Paraná | Breeding Lines | Carioca | Mesoamerican | G4 |
| Line363 | IDR-Paraná | Breeding Lines | Carioca | Mesoamerican | G2 |
| Line364 | IDR-Paraná | Breeding Lines | Carioca | Mesoamerican | G4 |
| Line365 | IDR-Paraná | Breeding Lines | Carioca | Mesoamerican | G4 |
| Line366 | IDR-Paraná | Breeding Lines | Carioca | Mesoamerican | G4 |
| Line367 | IDR-Paraná | Breeding Lines | Carioca | Mesoamerican | G4 |
| Line368 | IDR-Paraná | Breeding Lines | Carioca | Mesoamerican | G4 |
| Line369 | IDR-Paraná | Breeding Lines | Carioca | Mesoamerican | G4 |
| Line370 | IDR-Paraná | Breeding Lines | Carioca | Mesoamerican | G4 |
| Line371 | IDR-Paraná | Breeding Lines | Carioca | Mesoamerican | G4 |
| Line372 | IDR-Paraná | Breeding Lines | Carioca | Mesoamerican | G4 |
| Line373 | IDR-Paraná | Breeding Lines | Carioca | Mesoamerican | G2 |
| Line374 | IDR-Paraná | Breeding Lines | Carioca | Mesoamerican | G2 |
| Line375 | IDR-Paraná | Breeding Lines | Carioca | Mesoamerican | G2 |
| Line376 | IDR-Paraná | Breeding Lines | Black | Mesoamerican | G2 |
| Line377 | IDR-Paraná | Breeding Lines | Carioca | Mesoamerican | G2 |
| Line378 | IDR-Paraná | Breeding Lines | Carioca | Mesoamerican | G2 |
| Line379 | IDR-Paraná | Breeding Lines | Carioca | Mesoamerican | G2 |
| Line380 | IDR-Paraná | Breeding Lines | Carioca | Mesoamerican | G2 |
| Line381 | IDR-Paraná | Breeding Lines | Carioca | Mesoamerican | G2 |
| Line382 | IDR-Paraná | Breeding Lines | Carioca | Mesoamerican | G2 |
| Line383 | IDR-Paraná | Breeding Lines | Carioca | Mesoamerican | G2 |
| Line384 | IDR-Paraná | Breeding Lines | Carioca | Mesoamerican | G4 |
| Line385 | IDR-Paraná | Breeding Lines | Carioca | Mesoamerican | G4 |
| Line386 | IDR-Paraná | Breeding Lines | Carioca | Mesoamerican | G4 |
| Line387 | IDR-Paraná | Breeding Lines | Carioca | Mesoamerican | G4 |
| Line388 | IDR-Paraná | Breeding Lines | Carioca | Mesoamerican | G2 |
| Line389 | IDR-Paraná | Breeding Lines | Carioca | Mesoamerican | G2 |
| Line390 | IDR-Paraná | Breeding Lines | Carioca | Mesoamerican | G2 |
| Line391 | IDR-Paraná | Breeding Lines | Carioca | Mesoamerican | G2 |
| Line392 | IDR-Paraná | Breeding Lines | Carioca | Mesoamerican | G2 |
| Line393 | IDR-Paraná | Breeding Lines | Black | Mesoamerican | G2 |
| Line394 | IDR-Paraná | Breeding Lines | Carioca | Mesoamerican | G2 |
| Line395 | IDR-Paraná | Breeding Lines | Carioca | Mesoamerican | G2 |
| Line396 | IDR-Paraná | Breeding Lines | Carioca | Mesoamerican | G2 |
| Line397 | IDR-Paraná | Breeding Lines | Carioca | Mesoamerican | G2 |
| Line398 | IDR-Paraná | Breeding Lines | Carioca | Mesoamerican | G2 |
| Line399 | IDR-Paraná | Breeding Lines | Carioca | Mesoamerican | G2 |
| Line400 | IDR-Paraná | Breeding Lines | Carioca | Mesoamerican | G2 |
| Line401 | IDR-Paraná | Breeding Lines | Carioca | Mesoamerican | G2 |
| Line402 | IDR-Paraná | Breeding Lines | Black | Mesoamerican | G2 |
| Line403 | IDR-Paraná | Breeding Lines | Carioca | Mesoamerican | G2 |
| Line404 | IDR-Paraná | Breeding Lines | Carioca | Mesoamerican | G2 |
| Line405 | IDR-Paraná | Breeding Lines | Carioca | Mesoamerican | G2 |
| Line406 | IDR-Paraná | Breeding Lines | Carioca | Mesoamerican | G2 |
| Line407 | IDR-Paraná | Breeding Lines | Carioca | Mesoamerican | G2 |
| Line408 | IDR-Paraná | Breeding Lines | Carioca | Mesoamerican | G2 |
| Line409 | IDR-Paraná | Breeding Lines | Carioca | Mesoamerican | G2 |
| Line410 | IDR-Paraná | Breeding Lines | Carioca | Mesoamerican | G2 |
| Line411 | IDR-Paraná | Breeding Lines | Carioca | Mesoamerican | G2 |
| Line412 | IDR-Paraná | Breeding Lines | Carioca | Mesoamerican | G2 |
| Line413 | IDR-Paraná | Breeding Lines | Black | Mesoamerican | G2 |
| Line414 | IDR-Paraná | Breeding Lines | Carioca | Mesoamerican | G2 |
| Line415 | IDR-Paraná | Breeding Lines | Carioca | Mesoamerican | G2 |
| Line416 | IDR-Paraná | Breeding Lines | Carioca | Mesoamerican | G2 |
| Line417 | IDR-Paraná | Breeding Lines | Carioca | Mesoamerican | G2 |
| Line418 | IDR-Paraná | Breeding Lines | Carioca | Mesoamerican | G2 |
| Line419 | IDR-Paraná | Breeding Lines | Carioca | Mesoamerican | G2 |
| Line420 | IDR-Paraná | Breeding Lines | Carioca | Mesoamerican | G2 |
| Line421 | IDR-Paraná | Breeding Lines | Carioca | Mesoamerican | G2 |
| Line422 | IDR-Paraná | Breeding Lines | Carioca | Mesoamerican | G2 |
| Line423 | IDR-Paraná | Breeding Lines | Black | Mesoamerican | G2 |
| Line424 | IDR-Paraná | Breeding Lines | Carioca | Mesoamerican | G2 |
| Line425 | IDR-Paraná | Breeding Lines | Carioca | Mesoamerican | G2 |
| Line426 | IDR-Paraná | Breeding Lines | Carioca | Mesoamerican | G2 |
| Line427 | IDR-Paraná | Breeding Lines | Carioca | Mesoamerican | G2 |
| Line428 | IDR-Paraná | Breeding Lines | Carioca | Mesoamerican | G2 |
| Line429 | IDR-Paraná | Breeding Lines | Carioca | Mesoamerican | G2 |
| Line430 | IDR-Paraná | Breeding Lines | Carioca | Mesoamerican | G2 |
| Line431 | IDR-Paraná | Breeding Lines | Carioca | Mesoamerican | G2 |
| Line432 | IDR-Paraná | Breeding Lines | Black | Mesoamerican | G2 |
| Line433 | IDR-Paraná | Breeding Lines | Carioca | Mesoamerican | G2 |
| Line434 | IDR-Paraná | Breeding Lines | Carioca | Mesoamerican | G2 |
| Line435 | IDR-Paraná | Breeding Lines | Carioca | Mesoamerican | G2 |
| Line436 | IDR-Paraná | Breeding Lines | Carioca | Mesoamerican | G4 |
| Line437 | IDR-Paraná | Breeding Lines | Carioca | Mesoamerican | G2 |
| Line438 | IDR-Paraná | Breeding Lines | Carioca | Mesoamerican | G2 |
| Line439 | IDR-Paraná | Breeding Lines | Carioca | Mesoamerican | G2 |
| Line440 | IDR-Paraná | Breeding Lines | Carioca | Mesoamerican | G2 |
| Line441 | IDR-Paraná | Breeding Lines | Black | Mesoamerican | G2 |
| Line442 | IDR-Paraná | Breeding Lines | Carioca | Mesoamerican | G2 |
| Line443 | IDR-Paraná | Breeding Lines | Carioca | Mesoamerican | G2 |
| Line444 | IDR-Paraná | Breeding Lines | Carioca | Mesoamerican | G2 |
| Line445 | IDR-Paraná | Breeding Lines | Carioca | Mesoamerican | G2 |
| Line446 | IDR-Paraná | Breeding Lines | Carioca | Mesoamerican | G2 |
| Line447 | IDR-Paraná | Breeding Lines | Carioca | Mesoamerican | G2 |
| Line448 | IDR-Paraná | Breeding Lines | Carioca | Mesoamerican | G2 |
| Line449 | IDR-Paraná | Breeding Lines | Carioca | Mesoamerican | G2 |
| Line450 | IDR-Paraná | Breeding Lines | Black | Mesoamerican | G2 |
| Line451 | IDR-Paraná | Breeding Lines | Carioca | Mesoamerican | G2 |
| Line452 | IDR-Paraná | Breeding Lines | Carioca | Mesoamerican | G2 |
| Line453 | IDR-Paraná | Breeding Lines | Carioca | Mesoamerican | G2 |
| Line454 | IDR-Paraná | Breeding Lines | Carioca | Mesoamerican | G2 |
| Line455 | IDR-Paraná | Breeding Lines | Carioca | Mesoamerican | G2 |
| Line456 | IDR-Paraná | Breeding Lines | Carioca | Mesoamerican | G2 |
| Line457 | IDR-Paraná | Breeding Lines | Carioca | Mesoamerican | G4 |
| Line458 | IDR-Paraná | Breeding Lines | Carioca | Mesoamerican | G2 |
| Line459 | IDR-Paraná | Breeding Lines | Carioca | Mesoamerican | G3 |
| Line460 | IDR-Paraná | Breeding Lines | Black | Mesoamerican | G4 |
| Line461 | IDR-Paraná | Breeding Lines | Carioca | Mesoamerican | G2 |
| Line462 | IDR-Paraná | Breeding Lines | Carioca | Mesoamerican | G2 |
| Line463 | IDR-Paraná | Breeding Lines | Carioca | Mesoamerican | G2 |
| Line464 | IDR-Paraná | Breeding Lines | Carioca | Mesoamerican | G2 |
| Line465 | IDR-Paraná | Breeding Lines | Carioca | Mesoamerican | G3 |
| Line466 | IDR-Paraná | Breeding Lines | Carioca | Mesoamerican | G2 |
| Line467 | IDR-Paraná | Breeding Lines | Carioca | Mesoamerican | G2 |
| Line468 | IDR-Paraná | Breeding Lines | Carioca | Mesoamerican | G3 |
| Line469 | IDR-Paraná | Breeding Lines | Carioca | Mesoamerican | G2 |
| Line470 | IDR-Paraná | Breeding Lines | Carioca | Mesoamerican | G2 |
| Line471 | IDR-Paraná | Breeding Lines | Black | Mesoamerican | G2 |
| Line472 | IDR-Paraná | Breeding Lines | Carioca | Mesoamerican | G2 |
| Line473 | IDR-Paraná | Breeding Lines | Carioca | Mesoamerican | G2 |
| Line474 | IDR-Paraná | Breeding Lines | Carioca | Mesoamerican | G3 |
| Line475 | IDR-Paraná | Breeding Lines | Carioca | Mesoamerican | G2 |
| Line476 | IDR-Paraná | Breeding Lines | Carioca | Mesoamerican | G2 |
| Line477 | IDR-Paraná | Breeding Lines | Carioca | Mesoamerican | G2 |
| Line478 | IDR-Paraná | Breeding Lines | Carioca | Mesoamerican | G2 |
| Line479 | IDR-Paraná | Breeding Lines | Black | Mesoamerican | G2 |
| Line480 | IDR-Paraná | Breeding Lines | Carioca | Mesoamerican | G3 |
| Line481 | IDR-Paraná | Breeding Lines | Carioca | Mesoamerican | G3 |
| Line482 | IDR-Paraná | Breeding Lines | Carioca | Mesoamerican | G3 |
| Line483 | IDR-Paraná | Breeding Lines | Carioca | Mesoamerican | G2 |
| Line484 | IDR-Paraná | Breeding Lines | Carioca | Mesoamerican | G2 |
| Line485 | IDR-Paraná | Breeding Lines | Carioca | Mesoamerican | G2 |
| Line486 | IDR-Paraná | Breeding Lines | Carioca | Mesoamerican | G2 |
| Line487 | IDR-Paraná | Breeding Lines | Carioca | Mesoamerican | G2 |
| Line488 | IDR-Paraná | Breeding Lines | Carioca | Mesoamerican | G2 |
| Line489 | IDR-Paraná | Breeding Lines | Carioca | Mesoamerican | G2 |
| Line490 | IDR-Paraná | Breeding Lines | Black | Mesoamerican | G2 |
| Line491 | IDR-Paraná | Breeding Lines | Carioca | Mesoamerican | G2 |
| Line492 | IDR-Paraná | Breeding Lines | Carioca | Mesoamerican | G3 |
| Line493 | IDR-Paraná | Breeding Lines | Carioca | Mesoamerican | G2 |
| Line494 | IDR-Paraná | Breeding Lines | Carioca | Mesoamerican | G2 |
| Line495 | IDR-Paraná | Breeding Lines | Carioca | Mesoamerican | G5 |
| Line496 | IDR-Paraná | Breeding Lines | Carioca | Mesoamerican | G2 |
| Line497 | IDR-Paraná | Breeding Lines | Carioca | Mesoamerican | G2 |
| Line498 | IDR-Paraná | Breeding Lines | Carioca | Mesoamerican | G2 |
| Line499 | IDR-Paraná | Breeding Lines | Carioca | Mesoamerican | G3 |
| Line500 | IDR-Paraná | Breeding Lines | Carioca | Mesoamerican | G2 |
| Line501 | IDR-Paraná | Breeding Lines | Carioca | Mesoamerican | G2 |
| Line502 | IDR-Paraná | Breeding Lines | Carioca | Mesoamerican | G2 |
| Line503 | IDR-Paraná | Breeding Lines | Carioca | Mesoamerican | G2 |
| Line504 | IDR-Paraná | Breeding Lines | Carioca | Mesoamerican | G2 |
| Line505 | IDR-Paraná | Breeding Lines | Carioca | Mesoamerican | G2 |
| Line506 | IDR-Paraná | Breeding Lines | Carioca | Mesoamerican | G2 |
| Line507 | IDR-Paraná | Breeding Lines | Black | Mesoamerican | G2 |
| Line508 | IDR-Paraná | Breeding Lines | Black | Mesoamerican | G2 |
| Line509 | IDR-Paraná | Breeding Lines | Black | Mesoamerican | G2 |
| Line510 | IDR-Paraná | Breeding Lines | Black | Mesoamerican | G2 |
| Line511 | IDR-Paraná | Breeding Lines | Black | Mesoamerican | G2 |
| Line512 | IDR-Paraná | Breeding Lines | Black | Mesoamerican | G2 |
| Line513 | IDR-Paraná | Breeding Lines | Black | Mesoamerican | G2 |
| Line514 | IDR-Paraná | Breeding Lines | Black | Mesoamerican | G2 |
| Line515 | IDR-Paraná | Breeding Lines | Black | Mesoamerican | G2 |
| Line516 | IDR-Paraná | Breeding Lines | Black | Mesoamerican | G2 |
| Line517 | IDR-Paraná | Breeding Lines | Black | Mesoamerican | G2 |
| Line518 | IDR-Paraná | Breeding Lines | Black | Mesoamerican | G2 |
| Line519 | IDR-Paraná | Breeding Lines | Black | Mesoamerican | G2 |
| Line520 | IDR-Paraná | Breeding Lines | Black | Mesoamerican | G2 |
| Line521 | IDR-Paraná | Breeding Lines | Black | Mesoamerican | G2 |
| Line522 | IDR-Paraná | Breeding Lines | Black | Mesoamerican | G2 |
| Line523 | IDR-Paraná | Breeding Lines | Black | Mesoamerican | G2 |
| Line524 | IDR-Paraná | Breeding Lines | Black | Mesoamerican | G2 |
| Line525 | IDR-Paraná | Breeding Lines | Black | Mesoamerican | G3 |
| Line526 | IDR-Paraná | Breeding Lines | Black | Mesoamerican | G2 |
| Line527 | IDR-Paraná | Breeding Lines | Black | Mesoamerican | G2 |
| Line528 | IDR-Paraná | Breeding Lines | Black | Mesoamerican | G2 |
| Line529 | IDR-Paraná | Breeding Lines | Black | Mesoamerican | G3 |
| Line530 | IDR-Paraná | Breeding Lines | Black | Mesoamerican | G2 |
| Line531 | IDR-Paraná | Breeding Lines | Black | Mesoamerican | G2 |
| Line532 | IDR-Paraná | Breeding Lines | Black | Mesoamerican | G3 |
| Line533 | IDR-Paraná | Breeding Lines | Black | Mesoamerican | G2 |
| Line534 | IDR-Paraná | Breeding Lines | Black | Mesoamerican | G2 |
| Line535 | IDR-Paraná | Breeding Lines | Black | Mesoamerican | G2 |
| Line536 | IDR-Paraná | Breeding Lines | Black | Mesoamerican | G4 |
| Line537 | IDR-Paraná | Breeding Lines | Black | Mesoamerican | G4 |
| Line538 | IDR-Paraná | Breeding Lines | Black | Mesoamerican | G2 |
| Line539 | IDR-Paraná | Breeding Lines | Black | Mesoamerican | G2 |
| Line540 | IDR-Paraná | Breeding Lines | Black | Mesoamerican | G2 |
| Line541 | IDR-Paraná | Breeding Lines | Black | Mesoamerican | G2 |
| Line542 | IDR-Paraná | Breeding Lines | Black | Mesoamerican | G2 |
| Line543 | IDR-Paraná | Breeding Lines | Black | Mesoamerican | G2 |
| Line544 | IDR-Paraná | Breeding Lines | Black | Mesoamerican | G2 |
| Line545 | IDR-Paraná | Breeding Lines | Black | Mesoamerican | G2 |
| Line546 | IDR-Paraná | Breeding Lines | Black | Mesoamerican | G2 |
| Line547 | IDR-Paraná | Breeding Lines | Black | Mesoamerican | G2 |
| Line548 | IDR-Paraná | Breeding Lines | Black | Mesoamerican | G2 |
| Line549 | IDR-Paraná | Breeding Lines | Black | Mesoamerican | G2 |
| Line550 | IDR-Paraná | Breeding Lines | Black | Mesoamerican | G2 |
| Line551 | IDR-Paraná | Breeding Lines | Black | Mesoamerican | G2 |
| Line552 | IDR-Paraná | Breeding Lines | Black | Mesoamerican | G2 |
| Line553 | IDR-Paraná | Breeding Lines | Black | Mesoamerican | G2 |
| Line554 | IDR-Paraná | Breeding Lines | Black | Mesoamerican | G2 |
| Line555 | IDR-Paraná | Breeding Lines | Black | Mesoamerican | G2 |
| Line556 | IDR-Paraná | Breeding Lines | Black | Mesoamerican | G2 |
| Line557 | IDR-Paraná | Breeding Lines | Black | Mesoamerican | G2 |
| Line558 | IDR-Paraná | Breeding Lines | Black | Mesoamerican | G2 |
| Line559 | IDR-Paraná | Breeding Lines | Black | Mesoamerican | G4 |
| Line560 | IDR-Paraná | Breeding Lines | Black | Mesoamerican | G4 |
| Line561 | IDR-Paraná | Breeding Lines | Black | Mesoamerican | G4 |
| Line562 | IDR-Paraná | Breeding Lines | Black | Mesoamerican | G4 |
| Line563 | IDR-Paraná | Breeding Lines | Black | Mesoamerican | G4 |
| Line564 | IDR-Paraná | Breeding Lines | Black | Mesoamerican | G4 |
| Line565 | IDR-Paraná | Breeding Lines | Black | Mesoamerican | G4 |
| Line566 | IDR-Paraná | Breeding Lines | Black | Mesoamerican | G4 |
| Line567 | IDR-Paraná | Breeding Lines | Black | Mesoamerican | G1 |
| Line568 | IDR-Paraná | Breeding Lines | Carioca | Mesoamerican | G4 |
| Line569 | IDR-Paraná | Breeding Lines | Carioca | Mesoamerican | G4 |
| Line570 | IDR-Paraná | Breeding Lines | Carioca | Mesoamerican | G4 |
| Line571 | IDR-Paraná | Breeding Lines | Carioca | Mesoamerican | G4 |
| Line572 | IDR-Paraná | Breeding Lines | Carioca | Mesoamerican | G4 |
| Line573 | IDR-Paraná | Breeding Lines | Carioca | Mesoamerican | G4 |
| Line574 | IDR-Paraná | Breeding Lines | Carioca | Mesoamerican | G4 |
| Line575 | IDR-Paraná | Breeding Lines | Carioca | Mesoamerican | G4 |
| Line576 | IDR-Paraná | Breeding Lines | Carioca | Mesoamerican | G2 |
| Line577 | IDR-Paraná | Breeding Lines | Carioca | Mesoamerican | G2 |
| Line578 | IDR-Paraná | Breeding Lines | Carioca | Mesoamerican | G2 |
| Line579 | IDR-Paraná | Breeding Lines | Carioca | Mesoamerican | G2 |
| Line580 | IDR-Paraná | Breeding Lines | Carioca | Mesoamerican | G2 |
| Line581 | IDR-Paraná | Breeding Lines | Carioca | Mesoamerican | G2 |
| Line582 | IDR-Paraná | Breeding Lines | Carioca | Mesoamerican | G2 |
| Line583 | IDR-Paraná | Breeding Lines | Carioca | Mesoamerican | G2 |
| Line584 | IDR-Paraná | Breeding Lines | Carioca | Mesoamerican | G2 |
| Line585 | IDR-Paraná | Breeding Lines | Black | Mesoamerican | G4 |
| Line586 | IDR-Paraná | Breeding Lines | Black | Mesoamerican | G4 |
| Line587 | IDR-Paraná | Breeding Lines | Black | Mesoamerican | G4 |
| Line588 | IDR-Paraná | Breeding Lines | Black | Mesoamerican | G4 |
| Line589 | IDR-Paraná | Breeding Lines | Black | Mesoamerican | G4 |
| Line590 | IDR-Paraná | Breeding Lines | Black | Mesoamerican | G4 |
| Line591 | IDR-Paraná | Breeding Lines | Black | Mesoamerican | G4 |
| Line592 | IDR-Paraná | Breeding Lines | Black | Mesoamerican | G4 |
| Line593 | IDR-Paraná | Breeding Lines | Black | Mesoamerican | G4 |
| Line594 | IDR-Paraná | Breeding Lines | Carioca | Mesoamerican | G2 |
| Line595 | IDR-Paraná | Breeding Lines | Black | Mesoamerican | G4 |
| Line596 | IDR-Paraná | Breeding Lines | Black | Mesoamerican | G4 |
| Line597 | IDR-Paraná | Breeding Lines | Black | Mesoamerican | G4 |
| Line598 | IDR-Paraná | Breeding Lines | Black | Mesoamerican | G4 |
| Line599 | IDR-Paraná | Breeding Lines | Black | Mesoamerican | G4 |
| Line600 | IDR-Paraná | Breeding Lines | Black | Mesoamerican | G4 |
| Line601 | IDR-Paraná | Breeding Lines | Black | Mesoamerican | G4 |
| Line602 | IDR-Paraná | Breeding Lines | Black | Mesoamerican | G4 |
| Line603 | IDR-Paraná | Breeding Lines | Black | Mesoamerican | G4 |
| Line604 | IDR-Paraná | Breeding Lines | Black | Mesoamerican | G4 |
| Line605 | IDR-Paraná | Breeding Lines | Carioca | Mesoamerican | G2 |
| Line606 | IDR-Paraná | Breeding Lines | Black | Mesoamerican | G4 |
| Line607 | IDR-Paraná | Breeding Lines | Black | Mesoamerican | G4 |
| Line608 | IDR-Paraná | Breeding Lines | Black | Mesoamerican | G4 |
| Line609 | IDR-Paraná | Breeding Lines | Black | Mesoamerican | G4 |
| Line610 | IDR-Paraná | Breeding Lines | Black | Mesoamerican | G4 |
| Line611 | IDR-Paraná | Breeding Lines | Black | Mesoamerican | G4 |
| Line612 | IDR-Paraná | Breeding Lines | Black | Mesoamerican | G2 |
| Line613 | IDR-Paraná | Breeding Lines | Black | Mesoamerican | G4 |
| Line614 | IDR-Paraná | Breeding Lines | Black | Mesoamerican | G4 |
| Line615 | IDR-Paraná | Breeding Lines | Carioca | Mesoamerican | G2 |
| Line616 | IDR-Paraná | Breeding Lines | Black | Mesoamerican | G4 |
| Line617 | IDR-Paraná | Breeding Lines | Black | Mesoamerican | G2 |
| Line618 | IDR-Paraná | Breeding Lines | Black | Mesoamerican | G4 |
| Line619 | IDR-Paraná | Breeding Lines | Black | Mesoamerican | G4 |
| Line620 | IDR-Paraná | Breeding Lines | Black | Mesoamerican | G4 |
| Line621 | IDR-Paraná | Breeding Lines | Black | Mesoamerican | G4 |
| Line622 | IDR-Paraná | Breeding Lines | Carioca | Mesoamerican | G2 |
| Line623 | IDR-Paraná | Breeding Lines | Black | Mesoamerican | G4 |
| Line624 | IDR-Paraná | Breeding Lines | Black | Mesoamerican | G4 |
| Line625 | IDR-Paraná | Breeding Lines | Black | Mesoamerican | G4 |
| Line626 | IDR-Paraná | Breeding Lines | Black | Mesoamerican | G4 |
| Line627 | IDR-Paraná | Breeding Lines | Black | Mesoamerican | G4 |
| Line628 | IDR-Paraná | Breeding Lines | Black | Mesoamerican | G4 |
| Line629 | IDR-Paraná | Breeding Lines | Black | Mesoamerican | G4 |
| Line630 | IDR-Paraná | Breeding Lines | Black | Mesoamerican | G4 |
| Line631 | IDR-Paraná | Breeding Lines | Black | Mesoamerican | G4 |
| Line632 | IDR-Paraná | Breeding Lines | Black | Mesoamerican | G4 |
| Line633 | IDR-Paraná | Breeding Lines | Carioca | Mesoamerican | G2 |
| Line634 | IDR-Paraná | Breeding Lines | Black | Mesoamerican | G4 |
| Line635 | IDR-Paraná | Breeding Lines | Black | Mesoamerican | G4 |
| Line636 | IDR-Paraná | Breeding Lines | Black | Mesoamerican | G4 |
| Line637 | IDR-Paraná | Breeding Lines | Black | Mesoamerican | G4 |
| Line638 | IDR-Paraná | Breeding Lines | Black | Mesoamerican | G4 |
| Line639 | IDR-Paraná | Breeding Lines | Black | Mesoamerican | G4 |
| Line640 | IDR-Paraná | Breeding Lines | Black | Mesoamerican | G4 |
| Line641 | IDR-Paraná | Breeding Lines | Black | Mesoamerican | G4 |
| Line642 | IDR-Paraná | Breeding Lines | Carioca | Mesoamerican | G2 |
| Line643 | IDR-Paraná | Breeding Lines | Black | Mesoamerican | G4 |
| Line644 | IDR-Paraná | Breeding Lines | Black | Mesoamerican | G4 |
| Line645 | IDR-Paraná | Breeding Lines | Black | Mesoamerican | G4 |
| Line646 | IDR-Paraná | Breeding Lines | Black | Mesoamerican | G4 |
| Line647 | IDR-Paraná | Breeding Lines | Black | Mesoamerican | G4 |
| Line648 | IDR-Paraná | Breeding Lines | Black | Mesoamerican | G4 |
| Line649 | IDR-Paraná | Breeding Lines | Black | Mesoamerican | G4 |
| Line650 | IDR-Paraná | Breeding Lines | Black | Mesoamerican | G4 |
| Line651 | IDR-Paraná | Breeding Lines | Black | Mesoamerican | G4 |
| Line652 | IDR-Paraná | Breeding Lines | Carioca | Mesoamerican | G2 |
| Line653 | IDR-Paraná | Breeding Lines | Black | Mesoamerican | G4 |
| Line654 | IDR-Paraná | Breeding Lines | Black | Mesoamerican | G4 |
| Line655 | IDR-Paraná | Breeding Lines | Black | Mesoamerican | G4 |
| Line656 | IDR-Paraná | Breeding Lines | Black | Mesoamerican | G4 |
| Line657 | IDR-Paraná | Breeding Lines | Black | Mesoamerican | G4 |
| Line658 | IDR-Paraná | Breeding Lines | Black | Mesoamerican | G4 |
| Line659 | IDR-Paraná | Breeding Lines | Black | Mesoamerican | G4 |
| Line660 | IDR-Paraná | Breeding Lines | Black | Mesoamerican | G4 |
| Line661 | IDR-Paraná | Breeding Lines | Black | Mesoamerican | G4 |
| Line662 | IDR-Paraná | Breeding Lines | Carioca | Mesoamerican | G2 |
| Line663 | IDR-Paraná | Breeding Lines | Black | Mesoamerican | G4 |
| Line664 | IDR-Paraná | Breeding Lines | Black | Mesoamerican | G4 |
| Line665 | IDR-Paraná | Breeding Lines | Black | Mesoamerican | G4 |
| Line666 | IDR-Paraná | Breeding Lines | Black | Mesoamerican | G4 |
| Line667 | IDR-Paraná | Breeding Lines | Black | Mesoamerican | G4 |
| Line668 | IDR-Paraná | Breeding Lines | Black | Mesoamerican | G4 |
| Line669 | IDR-Paraná | Breeding Lines | Carioca | Mesoamerican | G2 |
| Line670 | IDR-Paraná | Breeding Lines | Black | Mesoamerican | G4 |
| Line671 | IDR-Paraná | Breeding Lines | Black | Mesoamerican | G4 |
| Line672 | IDR-Paraná | Breeding Lines | Black | Mesoamerican | G4 |
| Line673 | IDR-Paraná | Breeding Lines | Carioca | Mesoamerican | G2 |
| Line674 | IDR-Paraná | Breeding Lines | Carioca | Mesoamerican | G2 |
| Line675 | IDR-Paraná | Breeding Lines | Carioca | Mesoamerican | G2 |
| Line676 | IDR-Paraná | Breeding Lines | Carioca | Mesoamerican | G2 |
| Line677 | IDR-Paraná | Breeding Lines | Carioca | Mesoamerican | G2 |
| Line678 | IDR-Paraná | Breeding Lines | Carioca | Mesoamerican | G2 |
| Line679 | IDR-Paraná | Breeding Lines | Carioca | Mesoamerican | G2 |
| Line680 | IDR-Paraná | Breeding Lines | Carioca | Mesoamerican | G2 |
| Line681 | IDR-Paraná | Breeding Lines | Carioca | Mesoamerican | G2 |
| Line682 | IDR-Paraná | Breeding Lines | Carioca | Mesoamerican | G2 |
| Line683 | IDR-Paraná | Breeding Lines | Carioca | Mesoamerican | G2 |
| Line684 | IDR-Paraná | Breeding Lines | Carioca | Mesoamerican | G2 |
| Line685 | IDR-Paraná | Breeding Lines | Carioca | Mesoamerican | G2 |
| Line686 | IDR-Paraná | Breeding Lines | Carioca | Mesoamerican | G2 |
| Line687 | IDR-Paraná | Breeding Lines | Carioca | Mesoamerican | G2 |
| Line688 | IDR-Paraná | Breeding Lines | Carioca | Mesoamerican | G2 |
| Line689 | IDR-Paraná | Breeding Lines | Carioca | Mesoamerican | G2 |
| Line690 | IDR-Paraná | Breeding Lines | Carioca | Mesoamerican | G2 |
| Line691 | IDR-Paraná | Breeding Lines | Carioca | Mesoamerican | G2 |
| Line692 | IDR-Paraná | Breeding Lines | Carioca | Mesoamerican | G2 |
| Line693 | IDR-Paraná | Breeding Lines | Carioca | Mesoamerican | G2 |
| Line694 | IDR-Paraná | Breeding Lines | Carioca | Mesoamerican | G2 |
| Line695 | IDR-Paraná | Breeding Lines | Carioca | Mesoamerican | G2 |
| Line696 | IDR-Paraná | Breeding Lines | Carioca | Mesoamerican | G2 |
| Line697 | IDR-Paraná | Breeding Lines | Carioca | Mesoamerican | G2 |
| Line698 | IDR-Paraná | Breeding Lines | Carioca | Mesoamerican | G2 |
| Line699 | IDR-Paraná | Breeding Lines | Carioca | Mesoamerican | G2 |
| Line700 | IDR-Paraná | Breeding Lines | Carioca | Mesoamerican | G2 |
| Line701 | IDR-Paraná | Breeding Lines | Carioca | Mesoamerican | G2 |
| Line702 | IDR-Paraná | Breeding Lines | Carioca | Mesoamerican | G2 |
| Line703 | IDR-Paraná | Breeding Lines | Carioca | Mesoamerican | G2 |
| Line704 | IDR-Paraná | Breeding Lines | Carioca | Mesoamerican | G2 |
| Line705 | IDR-Paraná | Breeding Lines | Carioca | Mesoamerican | G2 |
| Line706 | IDR-Paraná | Breeding Lines | Carioca | Mesoamerican | G2 |
| Line707 | IDR-Paraná | Breeding Lines | Carioca | Mesoamerican | G2 |
| Line708 | IDR-Paraná | Breeding Lines | Carioca | Mesoamerican | G2 |
| Line709 | IDR-Paraná | Breeding Lines | Carioca | Mesoamerican | G2 |
| Line710 | IDR-Paraná | Breeding Lines | Carioca | Mesoamerican | G2 |
| Line711 | IDR-Paraná | Breeding Lines | Carioca | Mesoamerican | G2 |
| Line712 | IDR-Paraná | Breeding Lines | Carioca | Mesoamerican | G2 |
| Line713 | IDR-Paraná | Breeding Lines | Carioca | Mesoamerican | G2 |
| Line714 | IDR-Paraná | Breeding Lines | Carioca | Mesoamerican | G2 |
| Line715 | IDR-Paraná | Breeding Lines | Carioca | Mesoamerican | G2 |
| Line716 | IDR-Paraná | Breeding Lines | Carioca | Mesoamerican | G2 |
| Line717 | IDR-Paraná | Breeding Lines | Carioca | Mesoamerican | G3 |
| Line718 | IDR-Paraná | Breeding Lines | Carioca | Mesoamerican | G2 |
| Line719 | IDR-Paraná | Breeding Lines | Black | Mesoamerican | G4 |
| Line720 | IDR-Paraná | Breeding Lines | Black | Mesoamerican | G4 |
| Line721 | IDR-Paraná | Breeding Lines | Black | Mesoamerican | G4 |
| Line722 | IDR-Paraná | Breeding Lines | Black | Mesoamerican | G4 |
| Line723 | IDR-Paraná | Breeding Lines | Black | Mesoamerican | G2 |
| Line724 | IDR-Paraná | Breeding Lines | Black | Mesoamerican | G4 |
| Line725 | IDR-Paraná | Breeding Lines | Black | Mesoamerican | G4 |
| Line726 | IDR-Paraná | Breeding Lines | Black | Mesoamerican | G4 |
| Line727 | IDR-Paraná | Breeding Lines | Black | Mesoamerican | G2 |
| Line728 | IDR-Paraná | Breeding Lines | Black | Mesoamerican | G4 |
| Line729 | IDR-Paraná | Breeding Lines | Black | Mesoamerican | G4 |
| Line730 | IDR-Paraná | Breeding Lines | Black | Mesoamerican | G4 |
| Line731 | IDR-Paraná | Breeding Lines | Black | Mesoamerican | G2 |
| Line732 | IDR-Paraná | Breeding Lines | Black | Mesoamerican | G2 |
| Line733 | IDR-Paraná | Breeding Lines | Black | Mesoamerican | G2 |
| Line734 | IDR-Paraná | Breeding Lines | Black | Mesoamerican | G4 |
| Line735 | IDR-Paraná | Breeding Lines | Black | Mesoamerican | G4 |
| Line736 | IDR-Paraná | Breeding Lines | Black | Mesoamerican | G4 |
| Line737 | IDR-Paraná | Breeding Lines | Black | Mesoamerican | G4 |
| Line738 | IDR-Paraná | Breeding Lines | Black | Mesoamerican | G4 |
| Line739 | IDR-Paraná | Breeding Lines | Black | Mesoamerican | G4 |
| Line740 | IDR-Paraná | Breeding Lines | Black | Mesoamerican | G4 |
| Line741 | IDR-Paraná | Breeding Lines | Black | Mesoamerican | G4 |
| Line742 | IDR-Paraná | Breeding Lines | Black | Mesoamerican | G4 |
| Line743 | IDR-Paraná | Breeding Lines | Black | Mesoamerican | G4 |
| Line744 | IDR-Paraná | Breeding Lines | Black | Mesoamerican | G2 |
| Line745 | IDR-Paraná | Breeding Lines | Black | Mesoamerican | G4 |
| Line746 | IDR-Paraná | Breeding Lines | Black | Mesoamerican | G4 |
| Line747 | IDR-Paraná | Breeding Lines | Carioca | Mesoamerican | G2 |
| Line748 | IDR-Paraná | Breeding Lines | Carioca | Mesoamerican | G2 |
| Line749 | IDR-Paraná | Breeding Lines | Black | Mesoamerican | G2 |
| Line750 | IDR-Paraná | Breeding Lines | Carioca | Mesoamerican | G5 |
| Line751 | IDR-Paraná | Breeding Lines | Brown | Mesoamerican | G3 |
| Line752 | IDR-Paraná | Breeding Lines | Brown | Mesoamerican | G2 |
| Line753 | IDR-Paraná | Breeding Lines | Carioca | Mesoamerican | G5 |
| Line754 | IDR-Paraná | Breeding Lines | Brown | Mesoamerican | G3 |
| Line755 | IDR-Paraná | Breeding Lines | Brown | Mesoamerican | G3 |
| Line756 | IDR-Paraná | Breeding Lines | Brown | Mesoamerican | G3 |
| Line757 | IDR-Paraná | Breeding Lines | Carioca | Mesoamerican | G2 |
| Line758 | IDR-Paraná | Breeding Lines | Black | Mesoamerican | G2 |
| Line759 | IDR-Paraná | Breeding Lines | Brown | Mesoamerican | G2 |
| Line760 | IDR-Paraná | Breeding Lines | Brown | Mesoamerican | G3 |
| Line761 | IDR-Paraná | Breeding Lines | Brown | Mesoamerican | G2 |
| Line762 | IDR-Paraná | Breeding Lines | Brown | Mesoamerican | G2 |
| Line763 | IDR-Paraná | Breeding Lines | Brown | Mesoamerican | G2 |
| Line764 | IDR-Paraná | Breeding Lines | Brown | Mesoamerican | G4 |
| Line765 | IDR-Paraná | Breeding Lines | Brown | Mesoamerican | G2 |
| Line766 | IDR-Paraná | Breeding Lines | Brown | Mesoamerican | G2 |
| Line767 | IDR-Paraná | Breeding Lines | Brown | Mesoamerican | G2 |
| Line768 | IDR-Paraná | Breeding Lines | Brown | Mesoamerican | G2 |
| Line769 | IDR-Paraná | Breeding Lines | Brown | Mesoamerican | G2 |
| Line770 | IDR-Paraná | Breeding Lines | Brown | Mesoamerican | G2 |
| Line771 | IDR-Paraná | Breeding Lines | Carioca | Mesoamerican | G5 |
| Line772 | IDR-Paraná | Breeding Lines | Carioca | Mesoamerican | G2 |
| Line773 | IDR-Paraná | Breeding Lines | Carioca | Mesoamerican | G2 |
| Line774 | IDR-Paraná | Breeding Lines | Carioca | Mesoamerican | G2 |
| Line775 | IDR-Paraná | Breeding Lines | Brown | Mesoamerican | G2 |
| Line776 | IDR-Paraná | Breeding Lines | Carioca | Mesoamerican | G2 |
| Line777 | IDR-Paraná | Breeding Lines | Carioca | Mesoamerican | G2 |
| Line778 | IDR-Paraná | Breeding Lines | Brown | Mesoamerican | G4 |
| Line779 | IDR-Paraná | Breeding Lines | Brown | Mesoamerican | G2 |
| Line780 | IDR-Paraná | Breeding Lines | Brown | Mesoamerican | G5 |
| Line781 | IDR-Paraná | Breeding Lines | Carioca | Mesoamerican | G5 |
| Line782 | IDR-Paraná | Breeding Lines | Brown | Mesoamerican | G2 |
| Line783 | IDR-Paraná | Breeding Lines | Brown | Mesoamerican | G4 |
| Line784 | IDR-Paraná | Breeding Lines | Black | Mesoamerican | G2 |
| Line785 | IDR-Paraná | Breeding Lines | Carioca | Mesoamerican | G2 |
| Line786 | IDR-Paraná | Breeding Lines | Carioca | Mesoamerican | G5 |
| Line787 | IDR-Paraná | Breeding Lines | Black | Mesoamerican | G2 |
| Line788 | IDR-Paraná | Breeding Lines | Black | Mesoamerican | G2 |
| Line789 | IDR-Paraná | Breeding Lines | Black | Mesoamerican | G2 |
| Line790 | IDR-Paraná | Breeding Lines | Black | Mesoamerican | G2 |
| Line791 | IDR-Paraná | Breeding Lines | Black | Mesoamerican | G2 |
| Line792 | IDR-Paraná | Breeding Lines | Brown | Mesoamerican | G3 |
| Line793 | IDR-Paraná | Breeding Lines | Brown | Mesoamerican | G3 |
| Line794 | IDR-Paraná | Breeding Lines | Black | Mesoamerican | G2 |
| Line795 | IDR-Paraná | Breeding Lines | Carioca | Mesoamerican | G5 |
| Line796 | IDR-Paraná | Breeding Lines | Black | Mesoamerican | G2 |
| Line797 | IDR-Paraná | Breeding Lines | Black | Mesoamerican | G2 |
| Line798 | IDR-Paraná | Breeding Lines | Black | Mesoamerican | G3 |
| Line799 | IDR-Paraná | Breeding Lines | Black | Mesoamerican | G2 |
| Line800 | IDR-Paraná | Breeding Lines | Brown | Mesoamerican | G3 |
| Line801 | IDR-Paraná | Breeding Lines | Brown | Mesoamerican | G2 |
| Line802 | IDR-Paraná | Breeding Lines | Brown | Mesoamerican | G2 |
| Line803 | IDR-Paraná | Breeding Lines | Carioca | Mesoamerican | G5 |
| Line804 | IDR-Paraná | Breeding Lines | Brown | Mesoamerican | G2 |
| Line805 | IDR-Paraná | Breeding Lines | Carioca | Mesoamerican | G2 |
| Line806 | IDR-Paraná | Breeding Lines | Carioca | Mesoamerican | G2 |
| Line807 | IDR-Paraná | Breeding Lines | Purple | Mesoamerican | G1 |
| Line808 | IDR-Paraná | Breeding Lines | Carioca | Mesoamerican | G5 |
| Line809 | IDR-Paraná | Breeding Lines | Brown | Mesoamerican | G2 |
| Line810 | IDR-Paraná | Breeding Lines | Brown | Mesoamerican | G2 |
| Line811 | IDR-Paraná | Breeding Lines | Brown | Mesoamerican | G2 |
| Line812 | IDR-Paraná | Breeding Lines | Brown | Mesoamerican | G3 |
| Line813 | IDR-Paraná | Breeding Lines | Brown | Mesoamerican | G3 |
| Line814 | IDR-Paraná | Breeding Lines | Brown | Mesoamerican | G2 |
| Line815 | IDR-Paraná | Breeding Lines | Brown | Mesoamerican | G2 |
| Line816 | IDR-Paraná | Breeding Lines | Carioca | Mesoamerican | G5 |
| Line817 | IDR-Paraná | Breeding Lines | Brown | Mesoamerican | G2 |
| Line818 | IDR-Paraná | Breeding Lines | Brown | Mesoamerican | G3 |
| Line819 | IDR-Paraná | Breeding Lines | Brown | Mesoamerican | G3 |
| Line820 | IDR-Paraná | Breeding Lines | Brown | Mesoamerican | G3 |
| Line821 | IDR-Paraná | Breeding Lines | Brown | Mesoamerican | G3 |
| Line822 | IDR-Paraná | Breeding Lines | Brown | Mesoamerican | G2 |
| Line823 | IDR-Paraná | Breeding Lines | Carioca | Mesoamerican | G2 |
| Line824 | IDR-Paraná | Breeding Lines | Brown | Mesoamerican | G3 |
| Line825 | IDR-Paraná | Breeding Lines | Brown | Mesoamerican | G3 |
| Line826 | IDR-Paraná | Breeding Lines | Brown | Mesoamerican | G4 |
| Line827 | IDR-Paraná | Breeding Lines | Brown | Mesoamerican | G2 |
| Line828 | IDR-Paraná | Breeding Lines | Carioca | Mesoamerican | G5 |
| Line829 | IDR-Paraná | Breeding Lines | Carioca | Mesoamerican | G2 |
| Line830 | IDR-Paraná | Breeding Lines | Brown | Mesoamerican | G4 |
| Line831 | IDR-Paraná | Breeding Lines | Brown | Mesoamerican | G4 |
| Line832 | IDR-Paraná | Breeding Lines | Carioca | Mesoamerican | G5 |
| Line833 | IDR-Paraná | Breeding Lines | Carioca | Mesoamerican | G2 |
| Line834 | IDR-Paraná | Breeding Lines | Brown | Mesoamerican | G4 |
| Line835 | IDR-Paraná | Breeding Lines | Black | Mesoamerican | G2 |
| Line836 | IDR-Paraná | Breeding Lines | Brown | Mesoamerican | G2 |
| Line837 | IDR-Paraná | Breeding Lines | Carioca | Mesoamerican | G5 |
| Line838 | IDR-Paraná | Breeding Lines | Carioca | Mesoamerican | G5 |
| Line839 | IDR-Paraná | Breeding Lines | Carioca | Mesoamerican | G2 |
| Line840 | IDR-Paraná | Breeding Lines | Brown | Mesoamerican | G2 |
| Line841 | IDR-Paraná | Breeding Lines | Brown | Mesoamerican | G2 |
| Line842 | IDR-Paraná | Breeding Lines | Carioca | Mesoamerican | G5 |
| Line843 | IDR-Paraná | Breeding Lines | Black Carioca | Mesoamerican | G2 |
| Line844 | IDR-Paraná | Breeding Lines | Carioca | Mesoamerican | G5 |
| Line845 | IDR-Paraná | Breeding Lines | Black Carioca | Mesoamerican | G2 |
| Line846 | IDR-Paraná | Breeding Lines | Black Carioca | Mesoamerican | G2 |
| Line847 | IDR-Paraná | Breeding Lines | Black Carioca | Mesoamerican | G2 |
| Line848 | IDR-Paraná | Breeding Lines | Black Carioca | Mesoamerican | G2 |
| Line849 | IDR-Paraná | Breeding Lines | Brown | Mesoamerican | G2 |
| Line850 | IDR-Paraná | Breeding Lines | Black Carioca | Mesoamerican | G2 |
| Line851 | IDR-Paraná | Breeding Lines | Black Carioca | Mesoamerican | G2 |
| Line852 | IDR-Paraná | Breeding Lines | Brown | Mesoamerican | G2 |
| Line853 | IDR-Paraná | Breeding Lines | Black Carioca | Mesoamerican | G2 |
| Line854 | IDR-Paraná | Breeding Lines | Black Carioca | Mesoamerican | G2 |
| Line855 | IDR-Paraná | Breeding Lines | Brown | Mesoamerican | G2 |
| Line856 | IDR-Paraná | Breeding Lines | Brown | Mesoamerican | G2 |
| Line857 | IDR-Paraná | Breeding Lines | Brown | Mesoamerican | G2 |
| Line858 | IDR-Paraná | Breeding Lines | Carioca | Mesoamerican | G5 |
| Line859 | IDR-Paraná | Breeding Lines | Carioca | Mesoamerican | G5 |
| Line860 | IDR-Paraná | Breeding Lines | Carioca | Mesoamerican | G5 |
| Line861 | IDR-Paraná | Breeding Lines | Brown | Mesoamerican | G2 |
| Line862 | IDR-Paraná | Breeding Lines | Brown | Mesoamerican | G2 |
| Line863 | IDR-Paraná | Breeding Lines | Brown | Mesoamerican | G2 |
| Line864 | IDR-Paraná | Breeding Lines | Brown | Mesoamerican | G3 |
| Line865 | IDR-Paraná | Breeding Lines | Brown | Mesoamerican | G4 |
| Line866 | IDR-Paraná | Breeding Lines | Brown | Mesoamerican | G2 |
| Line867 | IDR-Paraná | Breeding Lines | Brown | Mesoamerican | G2 |
| Line868 | IDR-Paraná | Breeding Lines | Brown | Mesoamerican | G2 |
| Line869 | IDR-Paraná | Breeding Lines | Black | Mesoamerican | G2 |
| Line870 | IDR-Paraná | Breeding Lines | Brown | Mesoamerican | G2 |
| Line871 | IDR-Paraná | Breeding Lines | Black | Mesoamerican | G2 |
| Line872 | IDR-Paraná | Breeding Lines | Black | Mesoamerican | G2 |
| Line873 | IDR-Paraná | Breeding Lines | Black | Mesoamerican | G3 |
| Line874 | IDR-Paraná | Breeding Lines | Brown | Mesoamerican | G2 |
| Line875 | IDR-Paraná | Breeding Lines | Carioca | Mesoamerican | G2 |
| Line876 | IDR-Paraná | Breeding Lines | Black | Mesoamerican | G2 |
| Line877 | IDR-Paraná | Breeding Lines | Yellow | Mesoamerican | G2 |
| Line878 | IDR-Paraná | Breeding Lines | Black | Mesoamerican | G3 |
| Line879 | IDR-Paraná | Breeding Lines | Black Carioca | Mesoamerican | G2 |
| Line880 | IDR-Paraná | Breeding Lines | Brown | Mesoamerican | G2 |
| Line881 | IDR-Paraná | Breeding Lines | Pink | Mesoamerican | G2 |
| Line882 | IDR-Paraná | Breeding Lines | Brown | Mesoamerican | G4 |
| Line883 | IDR-Paraná | Breeding Lines | Brown | Mesoamerican | G4 |
| Line884 | IDR-Paraná | Breeding Lines | Black | Mesoamerican | G2 |
| Line885 | IDR-Paraná | Breeding Lines | Black | Mesoamerican | G2 |
| Line886 | IDR-Paraná | Breeding Lines | Brown | Mesoamerican | G2 |
| Line887 | IDR-Paraná | Breeding Lines | Brown | Mesoamerican | G2 |
| Line888 | IDR-Paraná | Breeding Lines | Black | Mesoamerican | G2 |
| Line889 | IDR-Paraná | Breeding Lines | Black | Mesoamerican | G2 |
| Mexico222 | - | Cultivars | Red | Andean | G4 |
| Mexico29 | - | Cultivars | Black | Mesoamerican | G2 |
| Michingan | - | Cultivars | White | Mesoamerican | G2 |
| INT19 | Farmers | Landraces | Black | Mesoamerican | G2 |
| MOC54 | CIAT | Breeding Lines | Red | Mesoamerican | G4 |
| MOC55 | CIAT | Breeding Lines | Red | Mesoamerican | G4 |
| MOC58 | CIAT | Breeding Lines | Red | Mesoamerican | G4 |
| MOC60 | CIAT | Breeding Lines | Red | Mesoamerican | G4 |
| Moruna | IAC | Cultivars | Black | Mesoamerican | G3 |
| Moruna80 | IAC | Cultivars | Black | Mesoamerican | G3 |
| MRK45 | CIAT | Breeding Lines | Red | Andean | G4 |
| MUS18 | CIAT | Breeding Lines | Red | Mesoamerican | G2 |
| MUS48 | CIAT | Breeding Lines | Red | Mesoamerican | G2 |
| MUS49 | CIAT | Breeding Lines | Red | Mesoamerican | G2 |
| MUS52 | CIAT | Breeding Lines | Black | Mesoamerican | G3 |
| MUS71 | CIAT | Breeding Lines | Red | Mesoamerican | G2 |
| MUS80 | CIAT | Breeding Lines | Red | Mesoamerican | G2 |
| N81018 | CIAT | Breeding Lines | White | Mesoamerican | G2 |
| NAB42 | CIAT | Breeding Lines | Black | Mesoamerican | G2 |
| NAB87 | CIAT | Breeding Lines | Black | Mesoamerican | G3 |
| NAG156 | CIAT | Breeding Lines | Black | Mesoamerican | G2 |
| NAG170 | CIAT | Breeding Lines | Black | Mesoamerican | G3 |
| Negro Argel | - | Cultivars | Black | Mesoamerican | G3 |
| NEP172 | - | Breeding Lines | Black | Mesoamerican | G3 |
| O159 | - | Breeding Lines | White | Mesoamerican | G2 |
| Ouro Negro | Embrapa | Cultivars | Black | Mesoamerican | G3 |
| Perola | Embrapa | Cultivars | Carioca | Mesoamerican | G2 |
| PI313343 | USDA | Landraces | Black | Mesoamerican | G3 |
| PI313537 | USDA | Landraces | Black | Mesoamerican | G3 |
| Porrilo70 | CIAT | Cultivars | Black | Mesoamerican | G3 |
| Porrilo Sintético | CIAT | Cultivars | Black | Mesoamerican | G2 |
| PP1 | - | Breeding Lines | Black | Mesoamerican | G3 |
| PP10 | - | Breeding Lines | Black | Mesoamerican | G3 |
| PP10A | - | Breeding Lines | White | Mesoamerican | G3 |
| PP12 | - | Breeding Lines | Black | Mesoamerican | G3 |
| PP13 | - | Breeding Lines | Black | Mesoamerican | G3 |
| PP15 | - | Breeding Lines | White | Mesoamerican | G2 |
| PP19 | - | Breeding Lines | Black | Mesoamerican | G2 |
| PP2 | - | Breeding Lines | Black | Mesoamerican | G3 |
| PP26 | - | Breeding Lines | Black | Mesoamerican | G3 |
| PP6 | - | Breeding Lines | Black | Mesoamerican | G2 |
| PP7 | - | Breeding Lines | Black | Mesoamerican | G2 |
| PR710291 | - | Breeding Lines | Red | Mesoamerican | G2 |
| Line969 | IDR-Paraná | Breeding Lines | Black | Mesoamerican | G2 |
| Line970 | IDR-Paraná | Breeding Lines | Black | Mesoamerican | G2 |
| Line971 | IDR-Paraná | Breeding Lines | Black | Mesoamerican | G2 |
| Line972 | IDR-Paraná | Breeding Lines | Black | Mesoamerican | G2 |
| Line973 | IDR-Paraná | Breeding Lines | Black | Mesoamerican | G2 |
| Line974 | IDR-Paraná | Breeding Lines | Black | Mesoamerican | G2 |
| Line975 | IDR-Paraná | Breeding Lines | Black | Mesoamerican | G2 |
| Line976 | IDR-Paraná | Breeding Lines | Black | Mesoamerican | G2 |
| Line977 | IDR-Paraná | Breeding Lines | Black | Mesoamerican | G2 |
| Line978 | IDR-Paraná | Breeding Lines | Black | Mesoamerican | G2 |
| Line979 | IDR-Paraná | Breeding Lines | Black | Mesoamerican | G2 |
| Line980 | IDR-Paraná | Breeding Lines | Black | Mesoamerican | G2 |
| BRS Radiante | Embrapa | Cultivars | Pinto | Andean | G2 |
| Line890 | IDR-Paraná | Breeding Lines | Brown | Mesoamerican | G2 |
| Line891 | IDR-Paraná | Breeding Lines | Brown | Mesoamerican | G2 |
| Line892 | IDR-Paraná | Breeding Lines | Brown | Mesoamerican | G5 |
| Line893 | IDR-Paraná | Breeding Lines | Red | Mesoamerican | G1 |
| Line894 | IDR-Paraná | Breeding Lines | Brown | Mesoamerican | G2 |
| Line895 | IDR-Paraná | Breeding Lines | Black | Mesoamerican | G3 |
| Line896 | IDR-Paraná | Breeding Lines | Purple | Mesoamerican | G2 |
| Line897 | IDR-Paraná | Breeding Lines | Black | Mesoamerican | G2 |
| Line898 | IDR-Paraná | Breeding Lines | Black | Mesoamerican | G3 |
| Line899 | IDR-Paraná | Breeding Lines | Brown | Mesoamerican | G2 |
| Line900 | IDR-Paraná | Breeding Lines | Purple | Mesoamerican | G1 |
| Line901 | IDR-Paraná | Breeding Lines | Brown | Mesoamerican | G4 |
| Line902 | IDR-Paraná | Breeding Lines | Black | Mesoamerican | G3 |
| Line903 | IDR-Paraná | Breeding Lines | Black | Mesoamerican | G2 |
| Line904 | IDR-Paraná | Breeding Lines | Brown | Mesoamerican | G4 |
| Line905 | IDR-Paraná | Breeding Lines | Black | Mesoamerican | G2 |
| Line906 | IDR-Paraná | Breeding Lines | Black | Mesoamerican | G2 |
| Line907 | IDR-Paraná | Breeding Lines | Black | Mesoamerican | G3 |
| Line908 | IDR-Paraná | Breeding Lines | Brown | Mesoamerican | G4 |
| Line909 | IDR-Paraná | Breeding Lines | Brown | Mesoamerican | G4 |
| Line910 | IDR-Paraná | Breeding Lines | Brown | Mesoamerican | G1 |
| Line911 | IDR-Paraná | Breeding Lines | Brown | Mesoamerican | G4 |
| Line912 | IDR-Paraná | Breeding Lines | Brown | Mesoamerican | G4 |
| Line913 | IDR-Paraná | Breeding Lines | Brown | Mesoamerican | G4 |
| Line914 | IDR-Paraná | Breeding Lines | Black | Mesoamerican | G2 |
| Line915 | IDR-Paraná | Breeding Lines | Black | Mesoamerican | G2 |
| Line916 | IDR-Paraná | Breeding Lines | White | Mesoamerican | G2 |
| Line917 | IDR-Paraná | Breeding Lines | Black Carioca | Mesoamerican | G2 |
| Line918 | IDR-Paraná | Breeding Lines | Carioca | Mesoamerican | G2 |
| Line919 | IDR-Paraná | Breeding Lines | Carioca | Mesoamerican | G2 |
| Line920 | IDR-Paraná | Breeding Lines | Carioca | Mesoamerican | G5 |
| Line921 | IDR-Paraná | Breeding Lines | Carioca | Mesoamerican | G5 |
| Line922 | IDR-Paraná | Breeding Lines | Carioca | Mesoamerican | G5 |
| Line923 | IDR-Paraná | Breeding Lines | Black Carioca | Mesoamerican | G2 |
| Line924 | IDR-Paraná | Breeding Lines | Black | Mesoamerican | G2 |
| Line925 | IDR-Paraná | Breeding Lines | Black | Mesoamerican | G2 |
| Line926 | IDR-Paraná | Breeding Lines | Black | Mesoamerican | G2 |
| Line927 | IDR-Paraná | Breeding Lines | Carioca | Mesoamerican | G3 |
| Line928 | IDR-Paraná | Breeding Lines | Brown | Mesoamerican | G4 |
| Line929 | IDR-Paraná | Breeding Lines | Brown | Mesoamerican | G4 |
| Line930 | IDR-Paraná | Breeding Lines | Brown | Mesoamerican | G4 |
| Line931 | IDR-Paraná | Breeding Lines | Brown | Mesoamerican | G4 |
| Line932 | IDR-Paraná | Breeding Lines | Carioca | Mesoamerican | G5 |
| Line933 | IDR-Paraná | Breeding Lines | Brown | Mesoamerican | G2 |
| Line934 | IDR-Paraná | Breeding Lines | Brown | Mesoamerican | G2 |
| Line935 | IDR-Paraná | Breeding Lines | Black | Mesoamerican | G2 |
| Line936 | IDR-Paraná | Breeding Lines | Brown | Mesoamerican | G4 |
| Line937 | IDR-Paraná | Breeding Lines | Brown | Mesoamerican | G2 |
| Line938 | IDR-Paraná | Breeding Lines | Brown | Mesoamerican | G2 |
| Line939 | IDR-Paraná | Breeding Lines | Black | Mesoamerican | G2 |
| Line940 | IDR-Paraná | Breeding Lines | Black | Mesoamerican | G2 |
| Line941 | IDR-Paraná | Breeding Lines | Brown | Mesoamerican | G2 |
| Line942 | IDR-Paraná | Breeding Lines | Brown | Mesoamerican | G2 |
| Line943 | IDR-Paraná | Breeding Lines | Black | Mesoamerican | G2 |
| Line944 | IDR-Paraná | Breeding Lines | Black | Mesoamerican | G3 |
| Line945 | IDR-Paraná | Breeding Lines | Brown | Mesoamerican | G2 |
| Line946 | IDR-Paraná | Breeding Lines | Black | Mesoamerican | G2 |
| Line947 | IDR-Paraná | Breeding Lines | Black | Mesoamerican | G3 |
| Line948 | IDR-Paraná | Breeding Lines | Black | Mesoamerican | G3 |
| Line949 | IDR-Paraná | Breeding Lines | Brown | Mesoamerican | G2 |
| Line950 | IDR-Paraná | Breeding Lines | Carioca | Mesoamerican | G3 |
| Line951 | IDR-Paraná | Breeding Lines | Black | Mesoamerican | G3 |
| Line952 | IDR-Paraná | Breeding Lines | Black | Mesoamerican | G2 |
| Line953 | IDR-Paraná | Breeding Lines | Brown | Mesoamerican | G2 |
| Line954 | IDR-Paraná | Breeding Lines | Brown | Mesoamerican | G4 |
| Line955 | IDR-Paraná | Breeding Lines | Brown | Mesoamerican | G4 |
| Line956 | IDR-Paraná | Breeding Lines | Brown | Mesoamerican | G4 |
| Line957 | IDR-Paraná | Breeding Lines | Brown | Mesoamerican | G4 |
| Line958 | IDR-Paraná | Breeding Lines | Brown | Mesoamerican | G4 |
| Line959 | IDR-Paraná | Breeding Lines | Brown | Mesoamerican | G2 |
| Line960 | IDR-Paraná | Breeding Lines | Black | Mesoamerican | G2 |
| Line961 | IDR-Paraná | Breeding Lines | Black | Mesoamerican | G3 |
| Line962 | IDR-Paraná | Breeding Lines | Black | Mesoamerican | G2 |
| Line963 | IDR-Paraná | Breeding Lines | Black | Mesoamerican | G2 |
| Line964 | IDR-Paraná | Breeding Lines | Black | Mesoamerican | G2 |
| Line965 | IDR-Paraná | Breeding Lines | Brown | Mesoamerican | G2 |
| Line966 | IDR-Paraná | Breeding Lines | Brown | Mesoamerican | G2 |
| Line967 | IDR-Paraná | Breeding Lines | Black Carioca | Mesoamerican | G2 |
| Line968 | IDR-Paraná | Breeding Lines | Black | Mesoamerican | G2 |
| REC100 | CIAT | Breeding Lines | Black Carioca | Mesoamerican | G2 |
| INT20 | - | Landraces | Black | Mesoamerican | G2 |
| Rico 23 | - | Cultivars | Black | Mesoamerican | G2 |
| Rico23/10 | - | Cultivars | Black | Mesoamerican | G2 |
| Rio Doce | - | Cultivars | Carioca | Mesoamerican | G5 |
| Rio Iguaçu | - | Cultivars | Black | Mesoamerican | G2 |
| Rio Negro | IDR-Paraná | Cultivars | Black | Mesoamerican | G2 |
| Rio Pardo | - | Cultivars | Black | Mesoamerican | G3 |
| Rio Piquiri | IDR-Paraná | Cultivars | Brown | Mesoamerican | G3 |
| Rio Tibagi | IDR-Paraná | Cultivars | Black | Mesoamerican | G3 |
| Rio Vermelho | IDR-Paraná | Cultivars | Purple | Mesoamerican | G2 |
| RIZ47 | CIAT | Breeding Lines | Brown | Mesoamerican | G2 |
| RIZ57 | CIAT | Breeding Lines | Carioca | Mesoamerican | G5 |
| RIZ59 | CIAT | Breeding Lines | Brown | Mesoamerican | G5 |
| RIZ72 | CIAT | Breeding Lines | Red | Mesoamerican | G4 |
| RJR11 | - | Breeding Lines | White | Mesoamerican | G2 |
| RJR14 | - | Breeding Lines | White | Mesoamerican | G2 |
| RJR21 | - | Breeding Lines | White | Mesoamerican | G4 |
| RJR22 | - | Breeding Lines | White | Mesoamerican | G3 |
| INT21 | Farmers | Landraces | Pink | Mesoamerican | G3 |
| INT22 | Farmers | Landraces | Pink | Mesoamerican | G2 |
| RosinhaG1 | - | Cultivars | Pink | Mesoamerican | G1 |
| INT23 | Farmers | Landraces | Purple | Mesoamerican | G1 |
| INT24 | Farmers | Landraces | Black | Mesoamerican | G2 |
| INT25 | Farmers | Landraces | Purple | Mesoamerican | G1 |
| INT26 | Farmers | Landraces | Brown | Mesoamerican | G1 |
| INT27 | Farmers | Landraces | Purple | Mesoamerican | G1 |
| BRS Ruda | Embrapa | Cultivars | Carioca | Mesoamerican | G2 |
| Line969 | IDR-Paraná | Breeding Lines | Black | Mesoamerican | G2 |
| Line970 | IDR-Paraná | Breeding Lines | Black | Mesoamerican | G2 |
| Line971 | IDR-Paraná | Breeding Lines | Purple | Mesoamerican | G1 |
| Line972 | IDR-Paraná | Breeding Lines | Purple | Mesoamerican | G1 |
| BRS Safira | Embrapa | Cultivars | Pink | Mesoamerican | G2 |
| Line973 | IDR-Paraná | Breeding Lines | Pinto | Mesoamerican | G2 |
| Line974 | IDR-Paraná | Breeding Lines | Carioca | Mesoamerican | G2 |
| Line975 | IDR-Paraná | Breeding Lines | Carioca | Mesoamerican | G2 |
| Line976 | IDR-Paraná | Breeding Lines | Carioca | Mesoamerican | G4 |
| Line977 | IDR-Paraná | Breeding Lines | Black | Mesoamerican | G2 |
| Line978 | IDR-Paraná | Breeding Lines | Black | Mesoamerican | G2 |
| Line979 | IDR-Paraná | Breeding Lines | Black | Mesoamerican | G2 |
| TURRIALBA5 | - | Breeding Lines | Black | Mesoamerican | G3 |
| INT28 | Farmers | Landraces | Red | Mesoamerican | G1 |
| INT29 | Farmers | Landraces | Red | Mesoamerican | G1 |
| WAF111 | CIAT | Breeding Lines | White | Andean | G1 |
| WAF130 | CIAT | Breeding Lines | White | Andean | G1 |
| WAF141 | CIAT | Breeding Lines | White | Andean | G1 |
| WAF145 | CIAT | Breeding Lines | White | Andean | G2 |
| WAF21 | CIAT | Breeding Lines | Red | Mesoamerican | G2 |
| INT30 | - | Breeding Lines | White | Mesoamerican | G2 |
| XAN174 | CIAT | Breeding Lines | Black | Mesoamerican | G3 |
| XAN180 | CIAT | Breeding Lines | Black | Mesoamerican | G3 |
| XAN200 | CIAT | Breeding Lines | Black | Mesoamerican | G1 |
| XAN201 | CIAT | Breeding Lines | Black | Mesoamerican | G3 |
| XAN206 | CIAT | Breeding Lines | Black | Mesoamerican | G3 |
| XAN208 | CIAT | Breeding Lines | Black | Mesoamerican | G3 |
| XAN217 | CIAT | Breeding Lines | Black | Mesoamerican | G3 |
| XAN218 | CIAT | Breeding Lines | Black | Mesoamerican | G3 |
| XAN220 | CIAT | Breeding Lines | Black | Mesoamerican | G3 |
| XAN226 | CIAT | Breeding Lines | Black | Mesoamerican | G3 |
| XAN236 | CIAT | Breeding Lines | Black | Mesoamerican | G2 |
| XAN247 | CIAT | Breeding Lines | Red | Mesoamerican | G2 |
| XAN251 | CIAT | Breeding Lines | Red | Mesoamerican | G4 |
| XAN252 | CIAT | Breeding Lines | Red | Mesoamerican | G4 |
| XAN91 | CIAT | Breeding Lines | Brown | Mesoamerican | G2 |
| INT31 | - | Breeding Lines | Pinto | Andean | G4 |

^1^CIAT = International Center for Tropical Agriculture (Centro Internacional de Agricultura Tropical), EMBRAPA = Brazilian Agricultural Research Corporation (Empresa Brasileira de Pesquisa Agropecuária), IAC = Agronomic Institute of Campinas (Instituto Agronômico de Campinas), IDR-Paraná = Rural Development Institute of Paraná – IAPAR – EMATER (Instituto de desenvolvimento Rural do Paraná), USDA = United States Departmento of Agriculture, ESALQ = Universidade de São Paulo - Escola Superior de Agricultura “Luiz de Queiroz”.
