# Supplementary material for: Diversity of nutritional content in seeds of Brazilian common bean germplasm
Source: PLoS One. 2020 Sep 28;15(9):e0239263. doi: 10.1371/journal.pone.0239263 (PMC7521705; doi:10.1371/journal.pone.0239263)
Supplement: S1 File — (DOCX) [file pone.0239263.s003.docx]

Dear,

1. Please ensure that the author list and affiliations are correct on the title page of your manuscript, and that your author contributions, competing interests, and financial disclosure are correct as listed below. All of these sections will be indexed in PubMed and published by PLOS ONE as you have written them. Please email plosone@plos.org if any changes to this content need to be made.

Jessica Delfini:
Conceptualization
Formal analysis
Writing – original draft

Vânia Moda-Cirino:
Conceptualization
Funding acquisition
Project administration
Supervision
Writing – review & editing

José dos Santos Neto:
Data curation
Formal analysis
Writing – review & editing

Juliana Sawada Buratto:
Conceptualization
Investigation

Paulo Mauricio Ruas:
Writing – review & editing

Please see here for the full list and definition of contributor roles: http://journals.plos.org/plosone/s/authorship#loc-author-contributions

Please ensure that the Competing Interests and Financial Disclosure statements listed below are suitable for publication. These sections will be indexed in PubMed and published by PLOS ONE as you have written them. Please email plosone@plos.org if any changes to these statements need to be made.

Competing Interests:
he authors have declared that no competing interests exist.

Financial Disclosure:
The funders had no role in study design, data collection and analysis, decision to publish, or preparation of the manuscript.

**Answer: ok**

2. Please confirm that all information in your Funding Information is also present in your Financial Disclosure. Only the Financial Disclosure section will be published alongside your article to describe your funding.

Funding Information:

Instituto de Desenvolvimento Rural do Paraná – IAPAR – EMATER, 13.1.12.01.00.008, Dr. Vânia Moda-Cirino Ministério do Desenvolvimento Social e Combate à Fome, MDS 424/2007, Dr. Vânia Moda-Cirino Conselho Nacional de Desenvolvimento Científico e Tecnológico, 479740/2004-6, Dr. Vânia Moda-Cirino

**Answer: ok**

3. Author Paulo Maurício Ruasis listed as Paulo Mauricio Ruas in the online submission form; we are unsure which version is correct. If the name is incorrect in the manuscript, please correct this. If the name is incorrect in the submission form, please let us know so we can correct the author’s profile for you. Please note that the use of middle initials/names should be consistent among the manuscript’s author list, the author list in the submission form, and the Author Contributions initials.

**Answer:** The correct name is **Paulo Mauricio Ruas**

4. Please clarify the affiliation of all authors Please refer to our downloadable sample files to make sure that your affiliations and byline are formatted correctly: <http://journals.plos.org/plosone/s/file?id=ba62/PLOSOne_formatting_sample_title_authors_affiliations.pdf>

**Answer: ok**

5. To prevent production delays, we recommend using the Author Formatting Checklist to confirm that your paper meets PLOS ONE's typesetting requirements for References, Tables, and Figures: <http://journals.plos.org/plosone/s/file?id=c819/plos-one-author-formatting-checklist.docx>.

This checklist is a reference tool for you; please do not upload the completed Author Formatting Checklist with your submission files.

**Answer: ok**

6. To ensure your figures meet our technical requirements, please run each figure included in your submission files through the PACE tool: <https://pacev2.apexcovantage.com/>. PACE will assess whether your figures meet our technical requirements and will fix the figure(s) or identify any problem(s) that cannot be automatically fixed. It can also convert figures to TIFF format, resize, and rename figures to meet our naming conventions.
To use PACE, first register as a user. Follow the instructions on the site for assessing and converting your figure files. If you experience any difficulty using this tool or have questions about any of the figures and/or images in your paper, please inform the journal office in your response letter.

**Answer:** The figures are attached.

CONFIDENTIAL: This email and any attachments are confidential and for the sole use of the individual(s) to whom they are addressed. If you have received this message in error please delete the message and notify [plosone@plos.org](mailto:plosone@plos.org).
